# Interaction between Wnt/β-catenin signaling pathway and EMT pathway mediates the mechanism of sunitinib resistance in renal cell carcinoma

**DOI:** 10.1186/s12885-024-11907-5

**Published:** 2024-02-05

**Authors:** Fangzhen Cai, Jianwei Li, Yanmei Zhang, Sihuai Huang, Wenbin Liu, Weifeng Zhuo, Chengzhi Qiu

**Affiliations:** 1 Department of Urology, The Second Affiliated Hospital of Fujian Medical University, Quanzhou, Fujian China; 2 Department of Urology, JinJiang Municipal Hospital, Quanzhou, Fujian China; 3 Department of General Surgery, The Second Affiliated Hospital of Fujian Medical University, Quanzhou, Fujian China

**Keywords:** Wnt, β-catenin, EMT, Sunitinib resistance, Renal cell carcinoma, Targeted therapy

## Abstract

**Background:**

Targeted drugs are the main methods of RCC treatment. However, drug resistance is common in RCC patients, in-depth study of the drug-resistant mechanism is essential.

**Methods:**

We constructed sunitinib resistant and Twist overexpressed A498 cells, and studied its mechanisms in vitro and in vivo.

**Results:**

In cell research, we found that either sunitinib resistance or Twist overexpression can activate Wnt/β-catenin and EMT signaling pathway, and the sunitinib resistance may work through β-catenin/TWIST/TCF4 trimer. In zebrafish research, we confirmed the similarity of Twist overexpression and sunitinib resistance, and the promoting effect of Twist overexpression on drug resistance.

**Conclusions:**

Sunitinib resistance and Twist overexpression can activate Wnt/β-catenin signaling pathway and EMT to promote the growth and metastasis of RCC cells.

**Supplementary Information:**

The online version contains supplementary material available at 10.1186/s12885-024-11907-5.

## Introduction

Renal cell carcinoma (RCC) is one of the most common malignant tumours of the urinary system, and its mortality ranks sixth in male tumours and eighth in female tumours [1, 2]. Due to the poor prognosis of early-stage RCC and resistance to both radiotherapy and chemotherapy, molecular targeted drugs have become the main methods of RCC treatment, and angiogenic receptor tyrosine kinase inhibitors (TKIs) remain the first-line therapy for RCC.

Sunitinib (SUTENT) is a multitarget tyrosine kinase receptor inhibitor that is a commonly used first-line drug for the treatment of RCC. It is an efficient inhibitor targeting the VEGF signalling pathway. It selectively inhibits PDGF-α/β, VEGFR-1/2/3, kit, FLT-3, CSF-1R and RET and has dual effects of killing tumour cells and inhibiting angiogenesis [2]. A recent global extended access trial showed that sunitinib can significantly prolong the progression-free survival times and overall survival times of patients [3]. However, 20% —30% of RCC patients are inherently resistant to sunitinib, and the remaining patients experience secondary resistance and disease progression after 6–15 months of treatment, resulting in the limited clinical efficacy of sunitinib [4]. Therefore, in-depth study of the drug-resistance mechanism of RCC is of great clinical significance to improve the therapeutic abilities for RCC.

Previous research results have shown that Wnt/β-catenin signalling pathway activation may be the key mechanism of sunitinib resistance [5–8]. The Wnt/β-catenin signalling pathway may have multiple node interactions with the EMT pathway, affecting tumour drug resistance. It has been shown that activating the Wnt/ beta-catenin signalling pathway affects breast cancer resistance to Sunitinib in breast cancer [9]. Therefore, we hypothesized that the Wnt/β-catenin signalling pathway cooperates with the EMT pathway to regulate sunitinib resistance in RCC. To verify this hypothesis, we constructed Sunitinib-resistant RCC cell lines to clarify the effects of Wnt/β-catenin signaling pathway and EMT pathway crossinuation on the mechanism of Sunitinib resistance in vitro and vivo. This study provides an experimental basis for the drug resistance mechanism of RCC to sunitinib.

## Methods

### Cell culture and sunitinib-resistant cell model induction

The human renal cell carcinoma cell line A498 (Xiamen ImmunoCell Biotechnology Co., Ltd.) was cultured in A498 cell-specific medium (Xiamen Immunocell Biotechnology Co., Ltd.). Sunitinib-resistant A498 cells were obtained by continuously exposing A498 cells to increasing concentrations of sunitinib, from 6 µM to 20 µM, over a period of > 12 weeks. All cells were cultured at 37 °C and 5% CO_2_ and were digested with trypsin-EDTA approximately twice a week. No mutagenic agents were used in the establishment of these sunitinib-resistant A498 (SR-A498) cells.

The resistance index (RI) was calculated according to the IC50 value.

### Determination of the proliferation of cells by CCK-8 assay

We incubated each group in MEM medium, 10% foetal bovine serum, 37 °C, and 5% CO_2_ for 0 h, 24 h, 48 h, 72 and 96 h, respectively, and added 10% CCK-8 reagent (10 µL). The culture was continued, and the OD value was measured at 450 nm after 1 h. The OD value indicates the cell proliferation, and the faster the cell proliferation, the higher the OD value.

### Determinaion of the migration ability of cells by cell scratch

We first incubated each group in a 37 °C 5% CO_2_ incubator for 4 h. When all cells adhered to the wall and reached confluency, tweezers were used to lift out the wound plug. Cells were washed with PBS 3 times, the scratched cells were removed, serum-free medium with mitomycin C (10 µg/mL, Sigma‒Aldrich) was added, and cells were replaced into a 37 °C 5% CO_2_ incubator. Following culture, photos were taken at 0 and 24 h, and the field of vision that contained “scratches” and cells on both sides at the same time under the microscope was selected for imaging. Before taking photos, the cells were washed with PBS and replaced in fresh serum-free medium.

### Determination of the migration ability of cells by BrdU incorporation assay

The cells were trypsinised and seeded on 6-well plates and cultured for 24 h. The cells were then treated with BrdU (10 µM) for 6 h. The BrdU incorporation rates of A498 and SR-A498 cells were measured with the BrdU Incorporation Assay Kit (Roche, IN) by in situ BrdU immunostaining according to the manufacturer’s instructions.

### Determination of migration and invasion ability by transwell assays

A total of 1 × 10^5^ cells/mL of cells were suspended in 2 mL of serum-free medium. Then, 100 µL of cells were added to the upper surface of the Transwell chamber. Subsequently, 600 µL of medium containing 20% FBS was added to the lower surface of the Transwell chamber and cultured at 37 °C for 24 h. After culturing for 24 h, the cells in the upper compartment were wiped off with a cotton swab. The medium in the chambers was removed, and 600 µL 4% paraformaldehyde was added to the lower chamber to fix the cells for 30 min. The paraformaldehyde was removed. Then, the cells that did not penetrate the membrane were wiped with a cotton swab. Then, 600 µL of 0.1% crystal violet (Sigma, Beijing, China) was added to the lower chamber for 15 min. For the Transwell invasion assays, 1 × 10^5^ cells/mL of cells were cultured in the Transwell chamber with extracellular matrix gel (Sigma, E1270), which was diluted on ice with serum-free medium at a ratio of 1:8. They were inoculated in a Transwell chamber and cultured in 10% FBS medium for 24–48 h. After culture, the Transwell chamber was removed. The excess cells in the chamber and the residual Matrigel were wiped with a cotton swab. After washing 3 times with PBS buffer, the cells were fixed with paraformaldehyde and stained with 0.1% crystal violet (Sigma, Beijing, China) using the lower surface of the membrane. The fields of view at 100× magnification (Olympus, CKX41) were counted and expressed as the average number of cells per field of view.

### Apoptosis level was determined by flow cytometry

Cell apoptosis was analysed by flow cytometry. The cells were inoculated in 6-well plates one day in advance, and then trypsin without EDTA was used for digestion and collection. Annexin V-Alexa 647 labelling treatment was performed according to the manufacturer’s instructions. Finally, flow cytometry analysis was performed to calculate the apoptosis rates of cells in each group.

### Western blot analysis

All cells were collected and incubated for 30 min in radioimmunoprecipitation assay lysis buffer (Sigma‒Aldrich; Merck KGaA) on ice and centrifuged at 15,000 x *g* for 5 min, and the total proteins in the supernatant were collected. The total protein concentration was determined by the RC-DC™ Protein Assay kit (Bio-Rad Laboratories, Inc.). The total proteins were diluted to 0.25 µg/µl in the presence of Laemmli 1x buffer (Cat. no. 161–0737; Bio-Rad Laboratories, Inc.) and β-mercaptoethanol. Then, the samples were denatured at 100 °C for 5 min, and 50 µl of sample was loaded onto an SDS‒PAGE gel (4–20% Mini-Protean® TGX Stain-Free™; Bio-Rad Laboratories, Inc.). After the target proteins were separated, the proteins were transferred to a PVDF membrane (0.22 μm PVDF; Millipore) through the Turbo™ Transfer system (Trans-Blot® Turbo™ Transfer system; Bio-Rad Laboratories, Inc.). The membrane was blocked with 5% skim milk diluted in PBS-0.1% Tween (PBS-T) for 1 h at room temperature and then incubated with primary anti-β-actin (1:5,000; Abcam), anti-Twist (1:500; Santa), anti-GSK3β (1:100; Affinity), anti-β-catenin (1:1,000; CST), anti-Snail (1:500; Santa), anti-E-cadherin (1:1,000; Proteintech), anti-TCF4 (1:1,000; Proteintech), anti-PCNA (1:1,000; Abcam), anti-Ki-67 (1:1,000; Proteintech), anti-MMP2 (1:1,000; Abcam), or anti-MMP9 (1:1,000; Abcam) antibodies in 5% bovine serum albumin (BSA; Sigma‒Aldrich; Merck KGaA) at 4 °C overnight. The membrane was then washed three times with PBS-T for 5 min and incubated with the corresponding secondary antibodies. Horseradish peroxidase-conjugated anti-rabbit or anti-mouse antibodies were diluted 1:3,500 in 5% BSA and incubated with the membrane for 1 h at room temperature. After three washes, HRP-ECL reagents (Santa Cruz Biotechnology, Inc.) were added to the membrane, and chemiluminescence was revealed using a Fuji LAS-3000 system (Fujifilm). The ratios of the blot density signal of specific protein bands to the control band were determined using ImageJ v1.51 software (National Institutes of Health).

### Coimmunoprecipitation assay

Target cells were collected, and the total cell lysates were prepared separately. Approximately 20 µl of cell lysate supernatant was left and boiled in 2x loading buffer for 5 min as the input group. The agarose beads were divided into new EP tubes in advance. A pipettor and pipette tip with the tip cut off were used to absorb the beads and ensure that the amount of beads in each tube is the same. The supernatant was carefully removed, and the antibody against protein A was added to the supernatant after cell lysis. Protein lysate (1 mg) was added to a 25 µl suspension, including 1:1 S beads and 2 µg of a protein antibody; 4 °C shaking table incubation was performed for 2–4 h. After binding, centrifugation was performed at 1400 rpm x 1 min, 4 °C. The supernatant was aspirated with a vacuum pump or pipettor, 800 µl NETN was added, mixed upside down, and centrifuged 3 times. The supernatant was discarded after the last time, the residual liquid was removed with a pipettor, 15 µl 2 x loading buffer was added to boil for 5 min as the co IP group sampling, and 10 µl 2 x loading buffer was added to cook the remaining sediment once again as the IP group sampling. Then, samples were collected in the order of coIP group, input1, marker, IP group and input2, and Western blotting analysis was carried out.

### Zebrafish experiment

After the embryos developed to 48 HPF, the fry were anaesthetised (using 3% MS222) and placed in the groove of the agar plate. Then, the stained cells were transplanted to the centre of the yolk sac of the zebrafish larvae using a microinjector. Each zebrafish was injected with 150–200 tumour cells. A group of at least 30 zebrafish were injected to ensure that 10 zebrafish were included in the group. Zebrafish fry transplanted with different human RCC A498 cell lines were treated with sunitinib at 1 µmol/L for 2 days. Then, the tumour cell fluorescence areas of 10 zebrafish in each group were counted and divided into four groups: Group I, which was transplanted with A498 cells named NC; Group II, which was transplanted with oe-Twist A498 cells named oe-Twist; Group III, which was transplanted with SR-A498 cells named SR-A498; and Group IV, which was transplanted with oe-Twist SR-A498 cells named SR-A498 + oe-Twist. At the two time points of 2 h (HPI) and 48 HPI after transplantation, 10 zebrafish in each group were photographed under a stereo fluorescence microscope, and the growth of tumour cells in zebrafish was observed and counted. This study was reviewed and approved by the ethics review board of The Second Affiliated Hospital, Fujian Medical University (No. [2021]51). All methods were carried out in accordance with relevant guidelines and regulations.

### Statistical analysis

All the experiments were independently performed at least three times, and all results are presented as the mean ± standard deviation. A t test was used to analyse the significant differences between groups. The fluorescence area of the zebrafish tumour was photographed by capture software with a body fluorescence microscope; all figures were completed on GraphPad; and the tumour fluorescence area was measured by ImageJ software. SPSS statistics software (version 23; IBM, Armonk, NY, USA) was used for statistical analysis. *P* ≤ 0.05 was deemed to be statistically significant.

## Results

### Establishment of a sunitinib-resistant A498 cell line

**Fig. 1:**
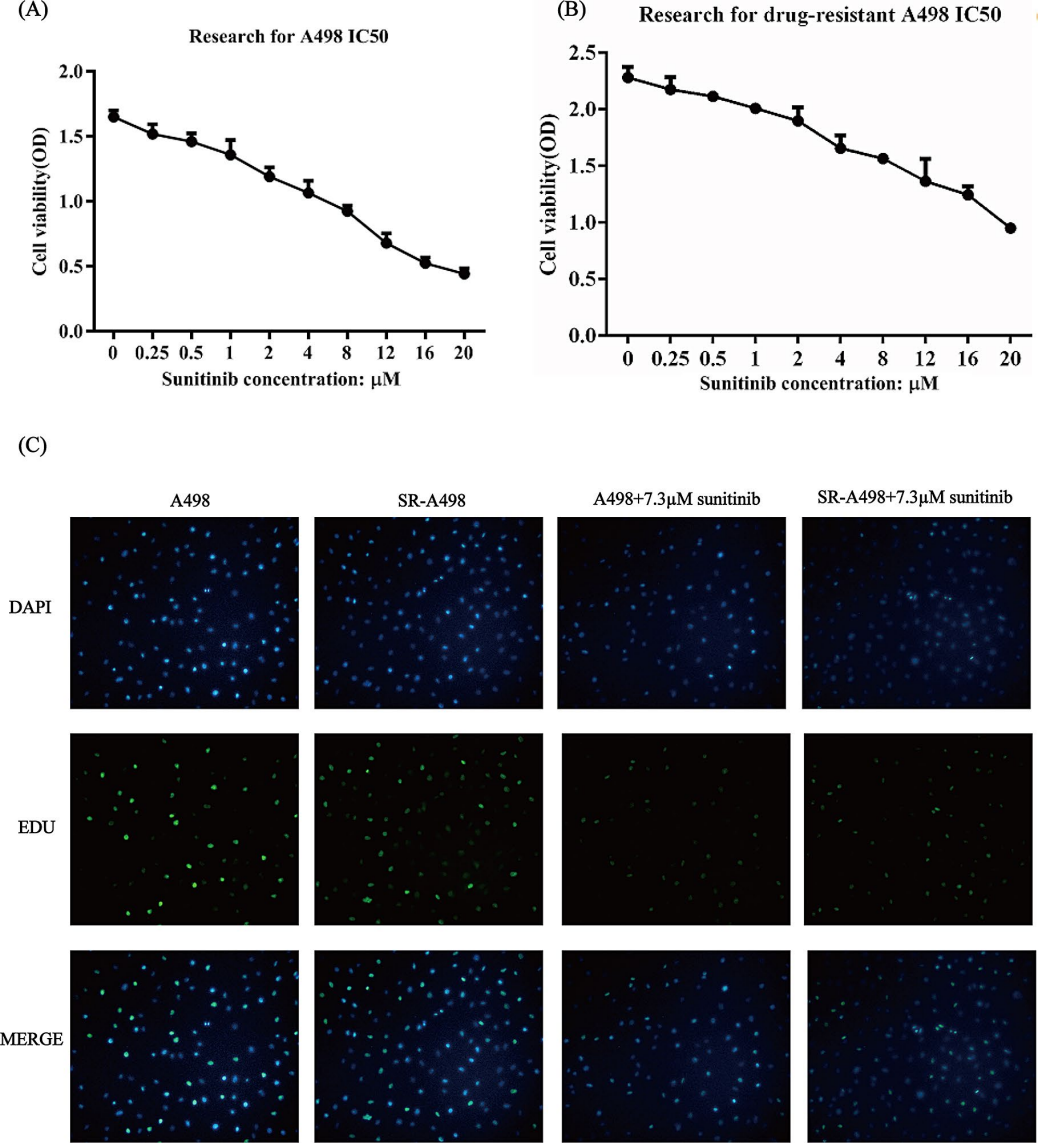
Established SR-A498 cells in vitro. A498 cells (**A**) and SR-A498 cells (**B**) proliferation under sunitinib exposure detected by CCK-8 assay, proliferation of A498 and SR-A498 cells by BrdU incorporation assay (**C**). All experiments were repeated in triplicate in three independent experiments

To construct the sunitinib-resistant A498 (SR-A498 for short) cell line, A498 cells were passaged in medium containing sunitnib from 6 µM to 20 µM for at least 12 weeks. Then, CCK-8 assays were performed to confirm the sunitinib sensitivities of the regular A498 cell line and SR-A498 cell line. The CCK-8 assay results showed that the IC_50_ of the parent strain (A498) (Fig. 1A) to sunitinib was 7.294 µM, while the IC_50_ of the constructed SR-A498 cells (Fig. 1B) to sunitinib was 18.114 µM. The drug resistance index (RI) was 2.5, and the BrdU incorporation assay showed that the cell viability of SR-A498 cells was higher than that of A498 cells, showing that SR-A498 cells have some resistance to sunitinib.

**Fig. 2:**
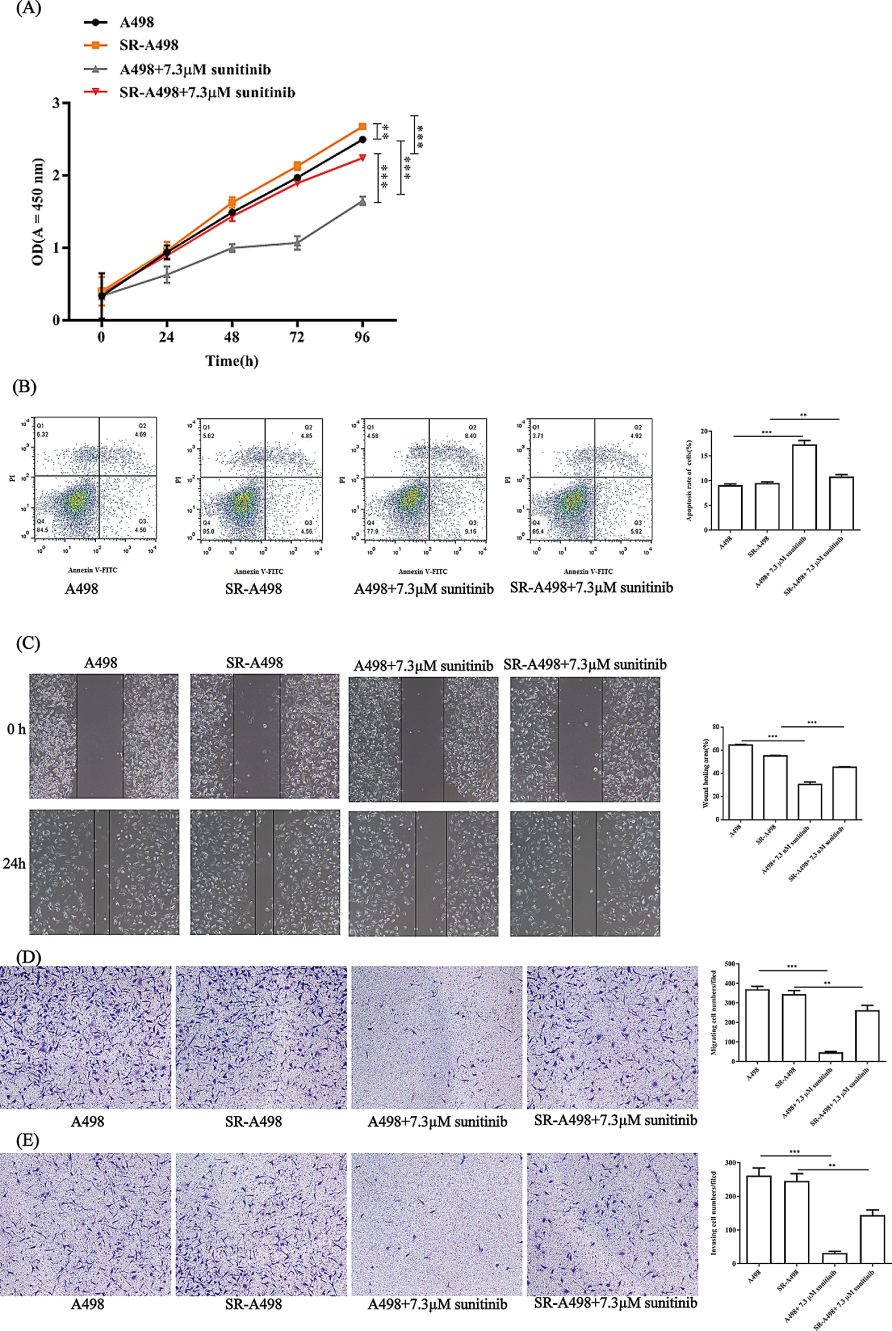
SR-A498 cell biological function in the presence/absence of sunitinib. The proliferation ability of SR-A498 cells was determined by CCK-8 assay (**A**). The figure only marks the significance of 96 h drug treatment. The apoptosis of A498 cells and SR-A498 cells was determined by flow cytometry (**B**). The migration ability of A498 cells and SR-A498 cells was determined by cell scratch experiments. (**C**). The migration (**D**) and invasion (**E**) abilities of A498 cells and SR-A498 cells were determined by Transwell assays. All representative images and statistical analyses are shown above. All experiments were repeated in triplicate in three independent experiments. *P < 0.05, **P < 0.01, ***P < 0.001

Then, cell function experiments were performed to verify the function of SR-A498. Cells were treated with the IC50 sunitinib concentration of A498 (7.294 µM of sunitinib). And it indicates that clinically resistant patients continue to take sunitinib for treatment. The results show that SR-A498 cells have a higher proliferation ability than A498 cells. After treatment with sunitinib, the proliferation of SR-A498 cells was not affected, while the proliferation of A498 cells was significantly inhibited. Flow cytometry showed different results, and the antiapoptotic ability of SR-A498 cells was significantly higher than that of A498 cells with or without sunitinib. For migration and invasiveness capability, SR-A498 cells also maintained a high invasiveness and migration capacity with or without 7.3 µM sunitinib treatment compared to the parent strain (Fig. 2C, D, E).

### SR-A498 cells overexpress twist and the construction of twist-overexpressing A498 cells

**Fig. 3:**
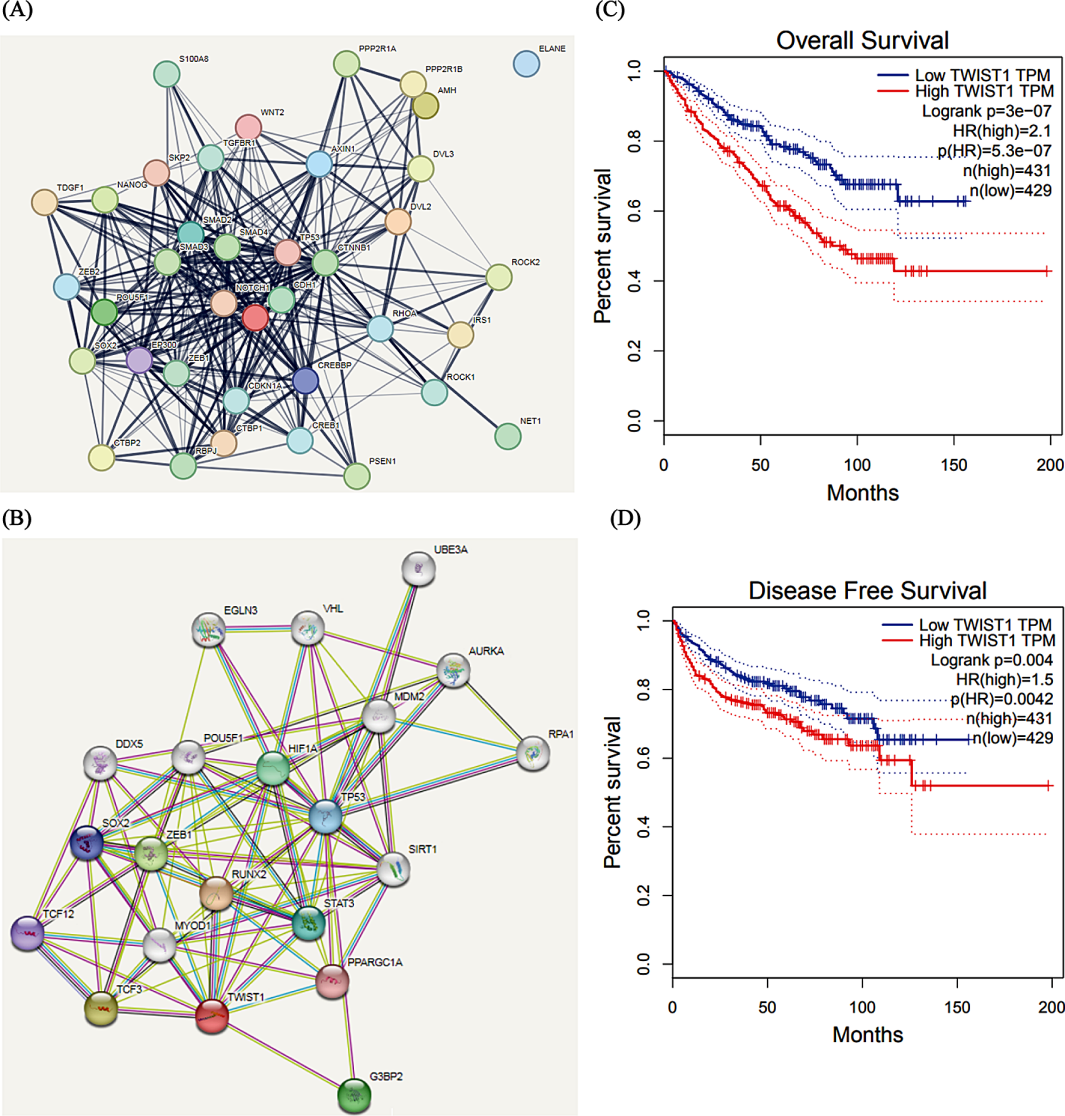
Twist is the key protein in the EMT and Wnt signalling pathway interaction. Predicted EMT (**A**) and Wnt (**B**) signalling pathway interacting proteins in the STRING database. Predicted Twist-interacting proteins in the STRING database. According to the GEPIA database, patients with high Twist expression have shorter OS (**C**) and PFS (**D**) times than those with low Twist expression

Recent comparative gene expression profiling of primary and metastatic RCC shows great differences in the TGF-β, Wnt/β-catenin and EMT signalling pathways [10] through data analysis in drug-resistant RCC. We searched for crosstalk genes in RCC and found the hub gene TWIST [8]. Twist not only affects both the Wnt/β-catenin and EMT signalling pathways but also has highly consistent predicted interacting proteins with EMT and Wnt signalling pathway interacting proteins (Fig. 3A, B). According to the GEPIA database (http://gepia.cancer-pku.cn/), patients with high Twist expression had obviously shorter progression-free survival (PFS) and overall survival (OS) times than those with low Twist expression (Fig. 3C, D).

**Fig. 4:**
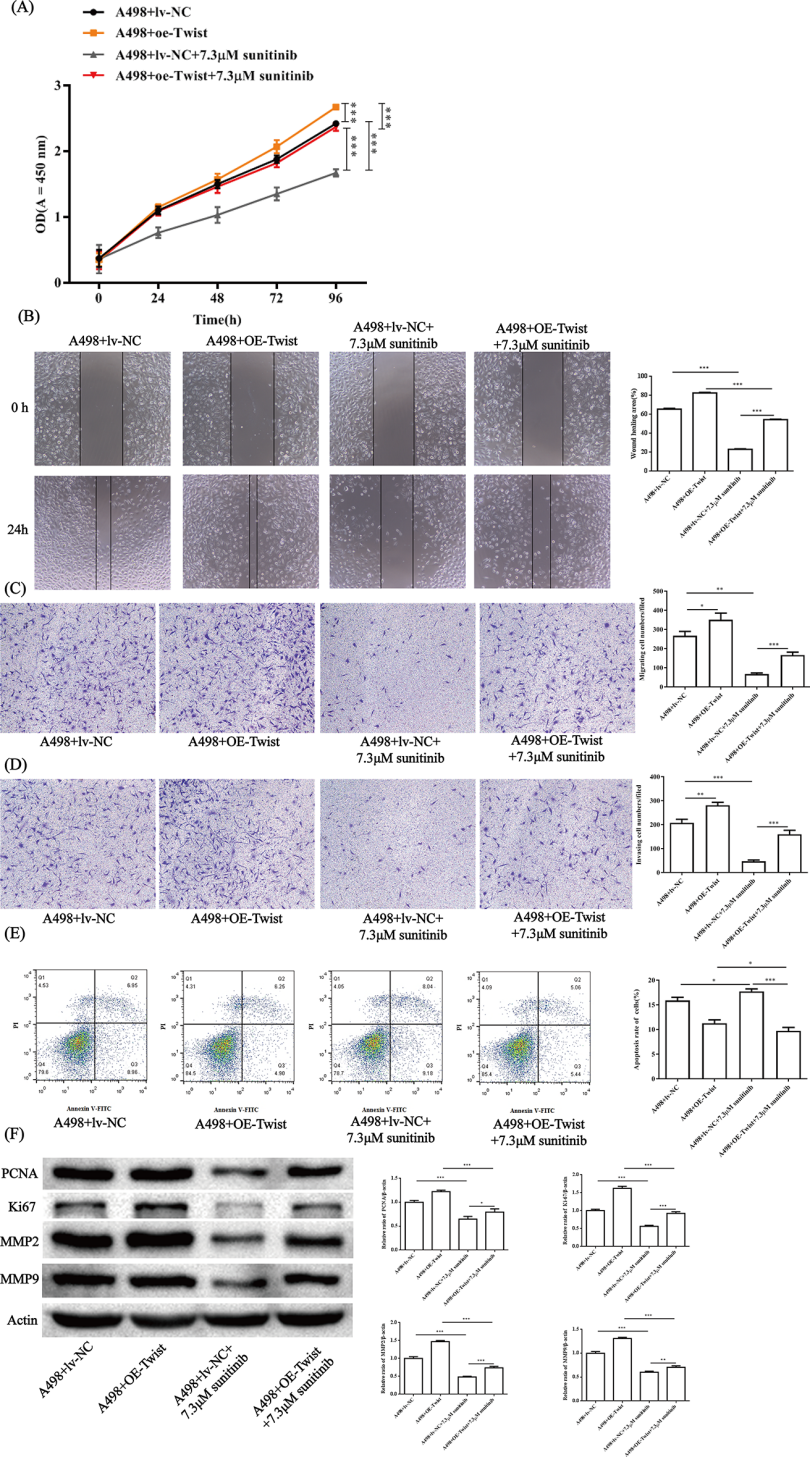
Biological function of Twist in A498 cells in the presence/absence of sunitinib. The proliferation ability of cells was determined by CCK-8 assay (**A**). The figure only marks the significance of 96 h drug treatment. The migration ability of cells was determined by a cell scratch experiment (**B**). The migration (**C**) and invasion (**D**) abilities of cells were determined by Transwell assays. Cell apoptosis was determined by flow cytometry (**E**). Key proteins related to proliferation, migration and invasion were detected by WB (**F**). All representative images and statistical analyses are shown above. All experiments were repeated in triplicate in three independent experiments. *P < 0.05, **P < 0.01, ***P < 0.001

In addition, in our SR-A498 cells, Twist was slightly overexpressed. The constructed Twist-overexpressing (oe-Twist) A498 cell line showed higher resistance to sunitinib in proliferation (Fig. 4A), migration (Fig. 4B, C), and invasiveness (Fig. 4D) ability than A498 cells transfected with the vector. The apoptosis of oe-Twist cells was inhibited compared to oe-NC in A498 cells (Fig. 4E). PCNA and Ki-67 are markers of cell proliferation, while MMP2 and MMP9 can reflect the level of cell migration and invasion. WB results showed that the protein expression levels of PCNA, Ki-67, MMP2 and MMP9 in oe-Twist were significantly higher than those in oe-NC, and the results were still consistent after sunitinib was added. The results showed that oe-Twist cells had some resistance to sunitinib (Fig. 4F).

### Twist overexpression exacerbates drug resistance in sunitinib-resistant strains

**Fig. 5:**
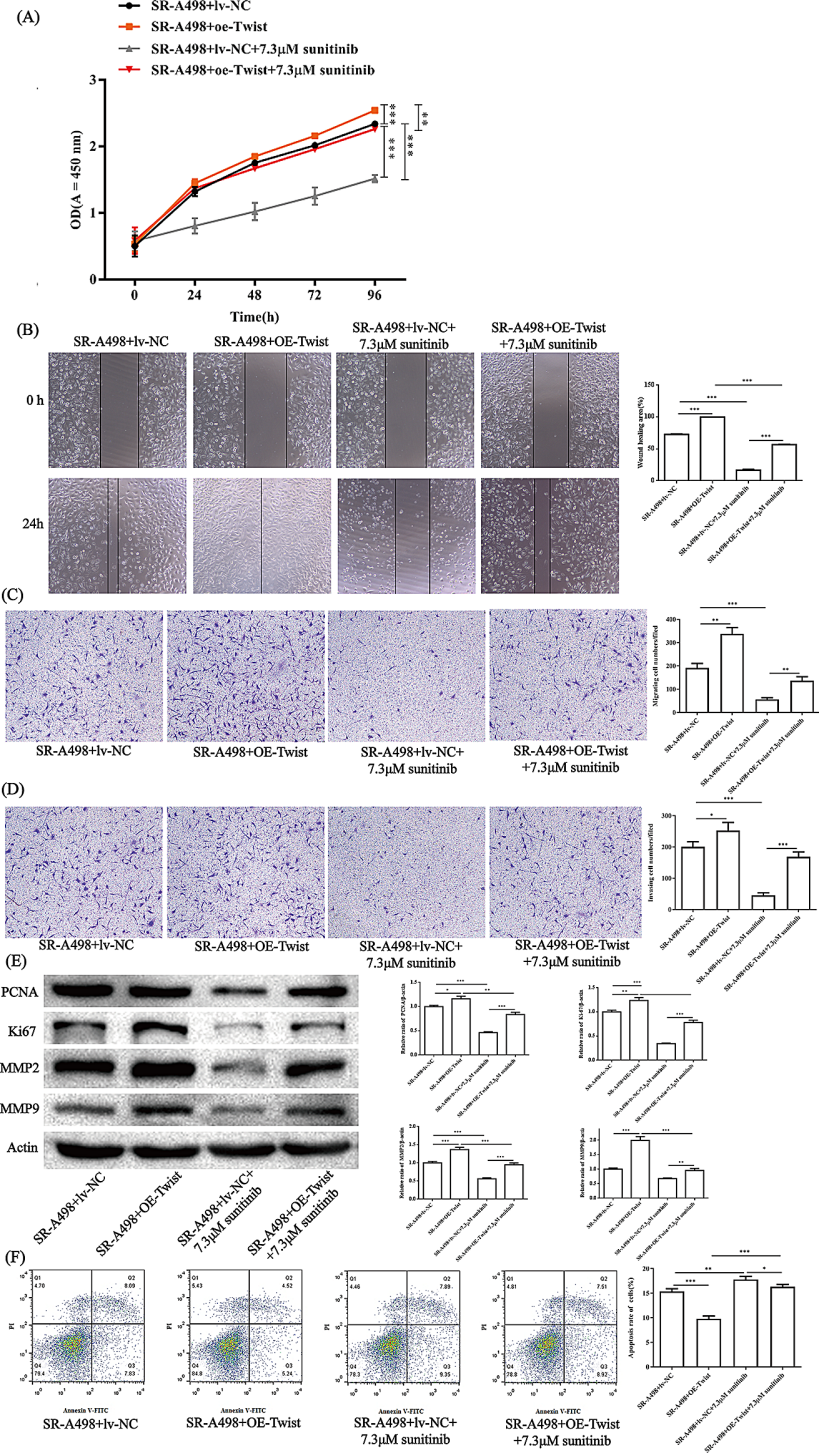
Twist overexpression in A498 cells basically eliminated the effect of sunitinib on SR-A498 cells. Determination of the proliferation ability of SR-A498 cells overexpressing Twist by CCK-8 assay (**A**). The results for the CCK-8 assay only mark the significance of 96 h drug treatment. Determination of the migration ability of SR-A498 cells overexpressing Twist by cell scratch. Representative images and statistical analysis (**B**). The migration (**C**) and invasion (**D**) abilities of cells were determined by Transwell assays. Key proteins related to proliferation, migration and invasion were detected by WB (**F**). Cell apoptosis was determined by flow cytometry (**E**). All experiments were repeated in triplicate in three independent experiments. *P < 0.05, **P < 0.01, ***P < 0.001

Through the detection of the proliferation and migration ability of SR-A498 cells overexpressing Twist, we found that overexpressing Twist in SR-A498 cells can enhance the proliferation, migration and invasion ability of SR-A498 cells (Fig. 5A–D). Then, the markers of proliferation, migration and invasion PCNA, Ki-67, MMP2 and MMP9 also showed the same results (Fig. 5E). Apoptosis was inhibited in the SR-A498 + lv-NC + 7.3 µM sunitinib group, while the SR-A498 + OE-Twist group showed a lower apoptosis rate (Fig. 5F). Other results showed that the sunitinib-treated oe-Twist SR-A498 group has no significant difference from the SR-A498 no treatment group (Fig. 5), which indicates that Twist overexpression basically eliminates the effect of sunitinib on the proliferation and migration of A498 cells.

### Effects of sunitinib resistance and twist overexpression on the Wnt/β-catenin pathway and EMT pathway

**Fig. 6:**
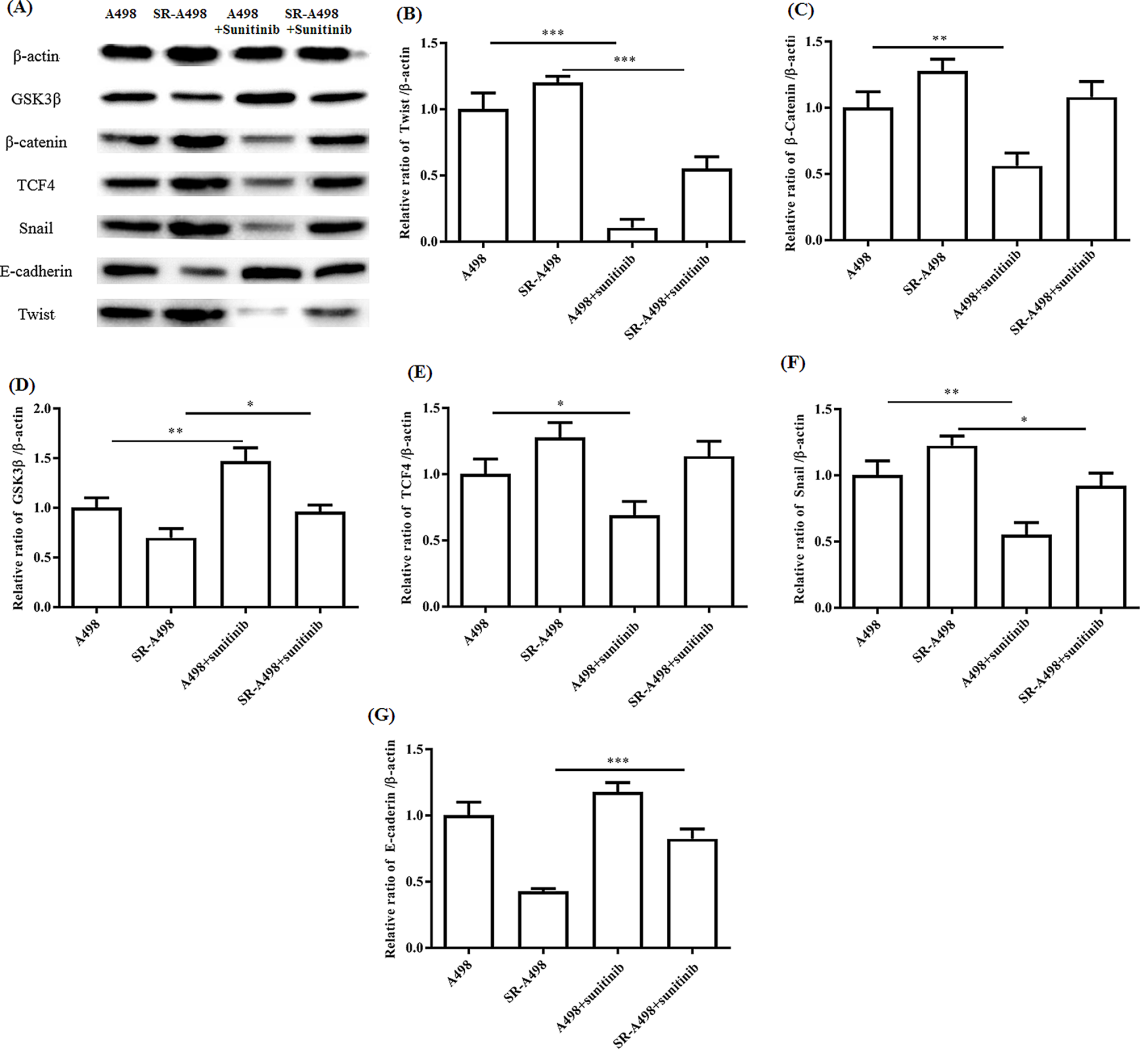
Analysis of the effect of sunitinib on the Wnt/β-catenin pathway and EMT pathway in A498 and SR-A498 cells. Representative WB results for sunitinib-treated A498 and SR-A498 cells (**A**). Relative Twist (**B**), β-catenin (**C**), GSK3β (**D**), TCF4 (**E**), Snail (**F**), and E-cadherin (**G**) expression to β-actin presented separately. In a series of WB analyses, at least three independent experiments were performed with similar results

**Fig. 7:**
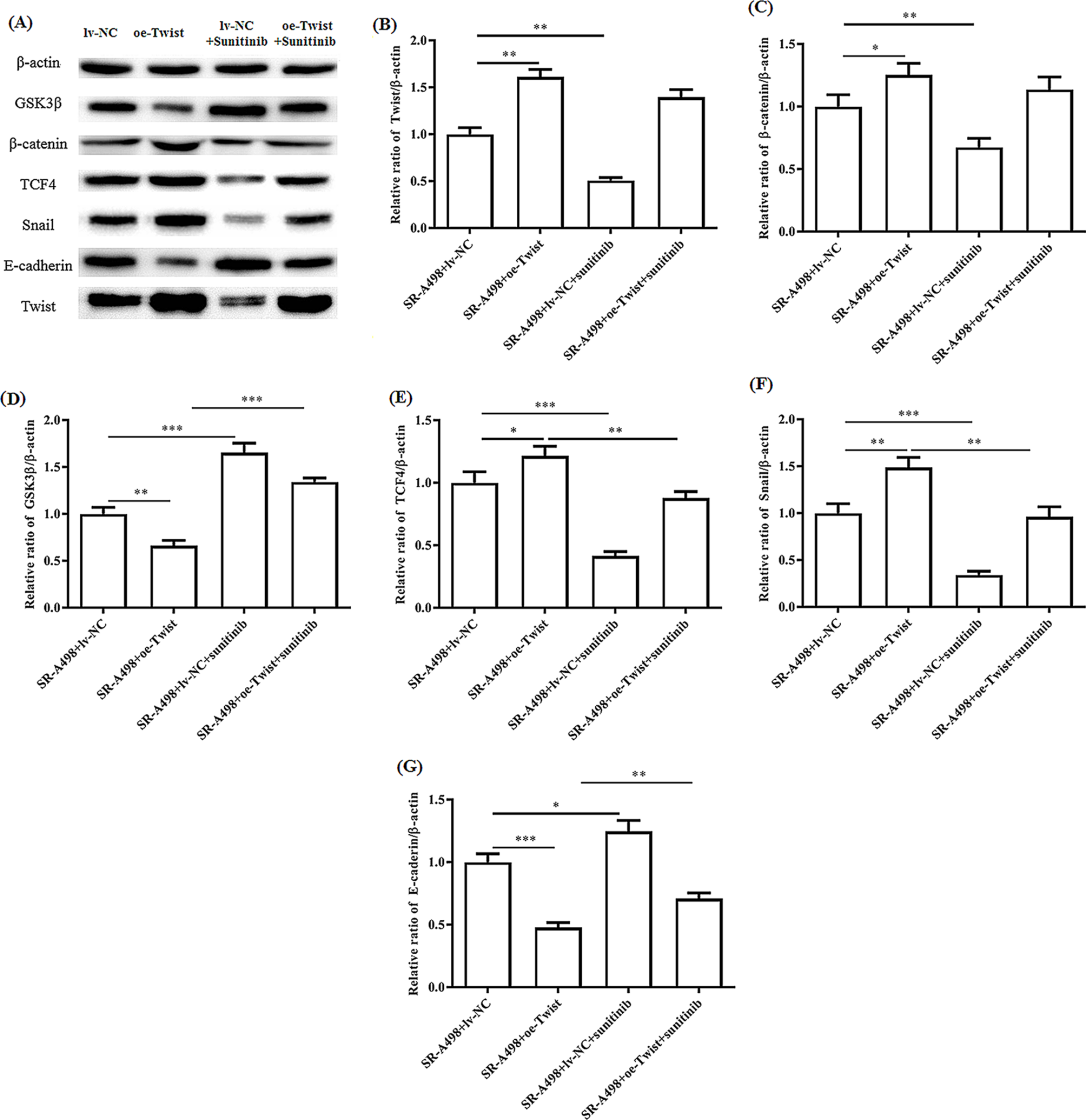
Analysis of the effect of sunitinib on the Wnt/β-catenin pathway and EMT pathway in Twist-overexpressing SR-A498 cells. Representative WB results for sunitinib-treated A498 and SR-A498 cells (**A**). Relative Twist (**B**), β-catenin (**C**), GSK3β (**D**), TCF4 (**E**), Snail (**F**), and E-cadherin (**G**) expression to β-actin presented separately. In a series of WB analyses, at least three independent experiments were performed with similar results

To investigate the molecular mechanism of sunitinib resistance and Twist overexpression on the Wnt/β-catenin pathway and EMT in renal cell carcinoma, we performed Western blot analysis to identify the differentially expressed proteins among SR-A498 cells, oe-Twist A498 cells, oe-Twist SR-A498 cells, and control cells. We found that compared with the control cells, either sunitinib-resistant or Twist-overexpressing cells can activate the Wnt/β-catenin signalling pathway, inhibit GSK-3β activity, upregulate the expression level of snail, reduce the expression of E-cadherin, and promote the occurrence of EMT and tumour metastasis, similar to both sunitinib resistance and Twist overexpression (Figs. 6 and 7). In addition, in the case of drug resistance and Twist overexpression, the continued use of normal doses of sunitinib still seems to have a certain effect on inhibiting tumour growth and metastasis.

### Effects of sunitinib resistance and twist overexpression on the interaction of β-catenin, TCF4 and twist

**Fig. 8:**
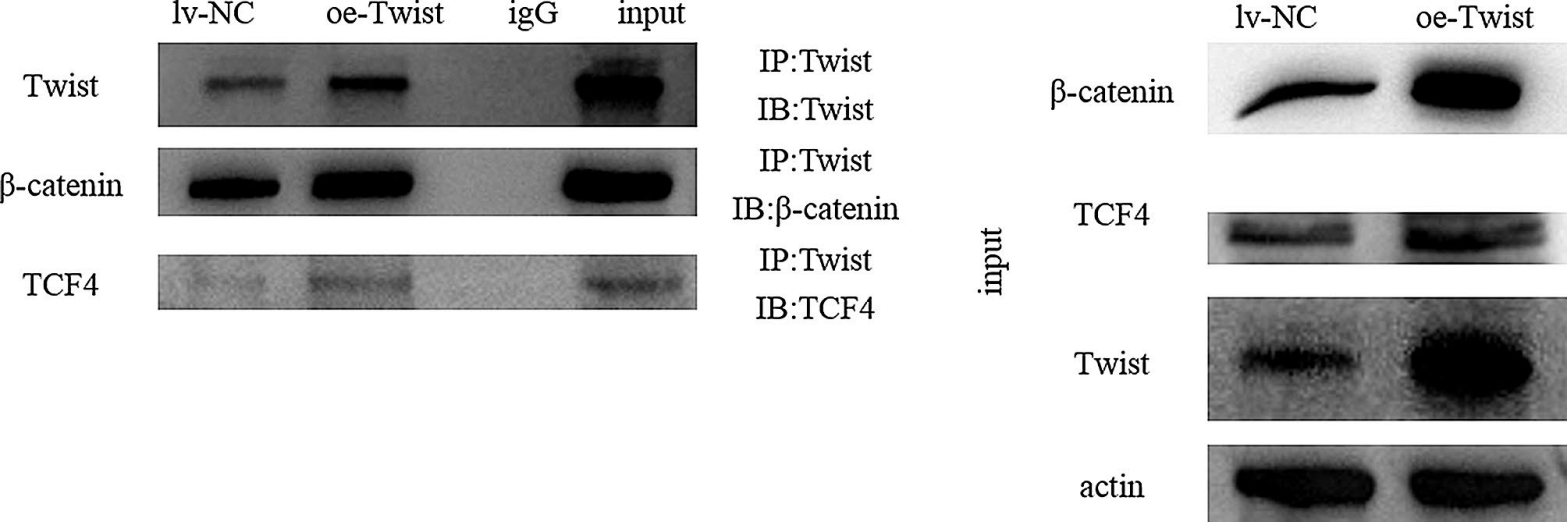
Twist interacts with β-catenin and TCF4 separately at the protein level as determined by co-IP. Co-IP using Twist nuclear lysates was performed with β-catenin and TCF4 or IgG antibodies

Western blot assays revealed the interactions between the Wnt/β-catenin pathway and the EMT pathway. Then, we further verified the interaction between Twist and β-catenin and Twist and TCF4 separately in A498 cells with Twist overexpression and sunitinib resistance in vitro by coimmunoprecipitation (co-IP) and luciferase assays. The results show the interactions between Twist and β-catenin, Twist and TCF4 at the protein level separately (Fig. 8); however, they did not show correlations at the transcription level (sup. Figure 1).

### In vivo zebrafish model verified the effect of twist overexpression

**Fig. 9:**
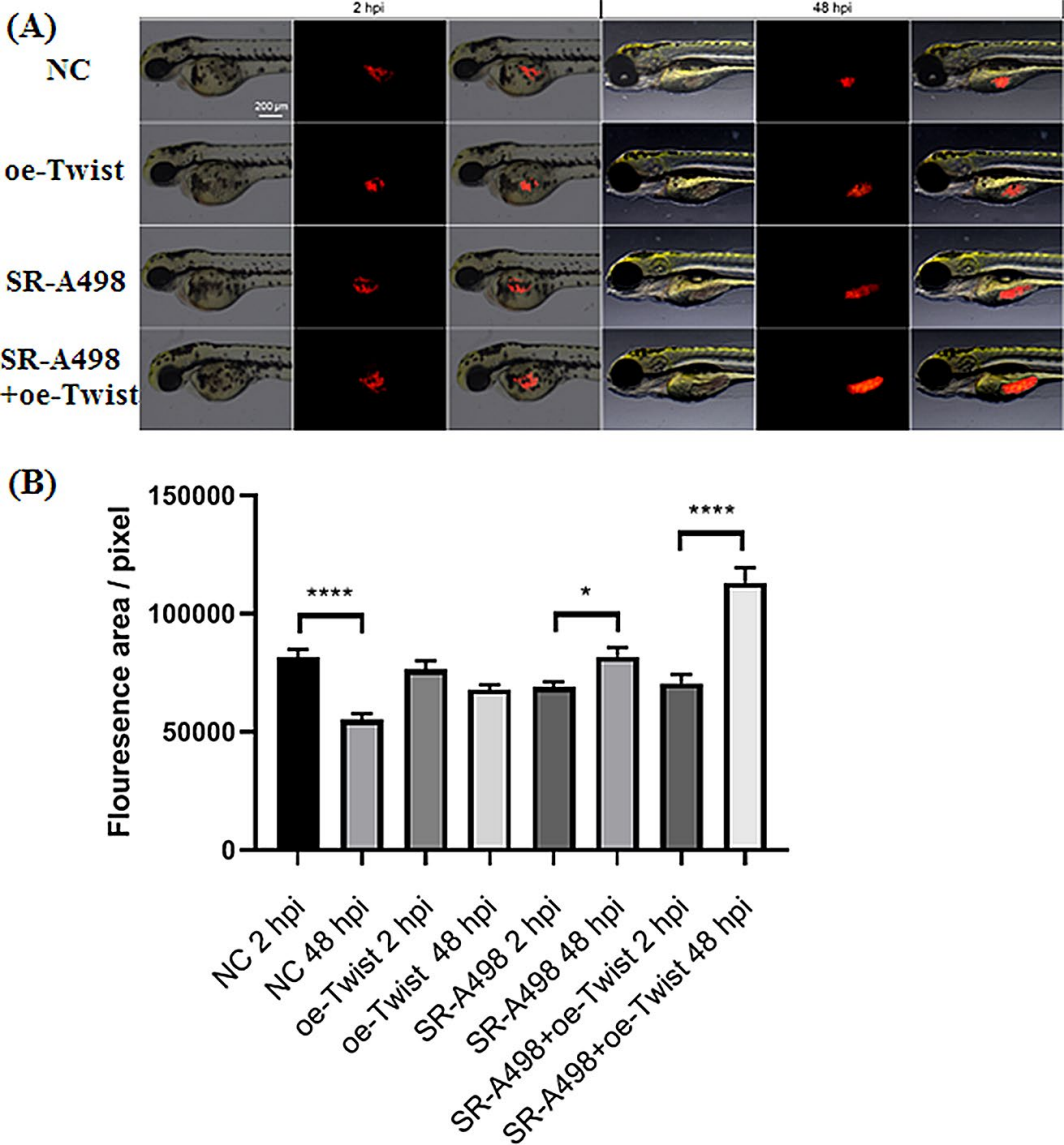
An in vivo zebrafish model verified the effect of Twist overexpression. Changes in the fluorescence area of tumour cells in different groups are shown in figure (**A**), and the tumour fluorescence area was measured by ImageJ (**B**)

Different groups of tumour cells were injected with PF zebrafish for 48 h and then treated with 1 µmol/L sunitinib. The reactions of cells in different groups to the drug significantly differed. Compared with the control groups, the fluorescence area of the oe-Twist group did not change significantly (*p* = 0.0573), the fluorescence area of the SR-A498 group increased significantly (*p* = 0.0113), and the fluorescence area of the SR-A498 + oe-Twist group increased most significantly (*P* < 0.0001) (Figs. 9).

## Discussion

EMT is a reversible developmental process. Cancer cells switch reversibly through EMT from an epithelial phenotype with acrobasal polarity and intercellular adhesion to a more active mesenchymal state with a spindle like morphology and anterior-to-anterior-to-distal polarity [11], and its characterized by increased metastatic and aggressive changes in cancer cells [12]. In the tumor microenvironment, tumor cell subpopulations can acquire stem cell characteristics through EMT, resulting in EMT-related drug resistance [11]. In our study, Sunitinib-resistant cell lines showed higher migration invasiveness, and detection of EMT-path-related proteins revealed that EMT transition to mesenchymal epithelium (MET) showed higher migration invasiveness. This transformation also increased the anti-apoptosis rate of cells [13]. In the experiment, the drug-resistant strains showed strong anti-apoptosis ability after the intervention of sunitinib. EMT involves a variety of signaling pathways, including transforming growth factor β (TGF-β), Wnt/β-catenin, etc. Activation of the Wnt/ beta-catenin pathway has been shown to be an important regulator of EMT in many different types of cancer [14]. Mutations or dysregulation of key proteins in the Wnt pathway can lead to many growth-related pathologies and tumorigenesis, tumor progression, and drug resistance [15]. Wnt is a kind of secretory glycoprotein that plays a role through autocrine or paracrine signalling. After secretion, Wnt can interact with specific receptors on the cell surface, causing β-catenin accumulation. β-catenin is a multifunctional protein that interacts with E-cadherin at the cell junction and participates in the formation of adhesive bands, while free β-catenin can enter the nucleus and regulate gene expression. Its abnormal expression or activation can trigger tumour development.Researchers at the University of Leeds in the UK confirmed that increasing VDR levels in melanoma cells can reduce the activity of the Wnt/β-catenin pathway, slow the growth of melanoma cells and prevent tumour cell metastasis to the lung [16]. In addition, runx1, which plays an important role as an oncogene and anticancer gene in epithelial tumours, has been found to promote the occurrence of colorectal cancer (CRC) by activating the Wnt/β-catenin signalling pathway and EMT to promote CRC metastasis [6]. In the drug resistance, inhibiting Wnt signaling can make cancer cells sensitive to chemotherapy. For example, inhibition of the Wnt/β-catenin pathway reverses multidrug resistance in NSCLC cells and in non-small cell lung cancer [17]. In myeloid leukemia cells, the Wnt inhibitor (FH535) makes them sensitive to the chemotherapy drug imatinib and enhances its chemotherapy effect [18]. A similar effect of Wnt inhibitors was observed in ovarian cancer cells, where inhibition of the Wnt pathway reveQpment of RCC.

Many researches shows that Wnt integrates with other signalling pathways to promote the occurrence and development of EMT. Both MAPK in the RTK/RAS/MAPK pathway and PKB in the PI3K/ILK/PKB pathway can inhibit GSK-3β active function [19, 20]. Therefore, these two pathways can interact with the Wnt/β-catenin signalling pathway through GSK-3β, upregulate the expression level of snail, reduce the expression of E-cadherin, and theoretically promote the occurrence of EMT and tumour metastasis. Wnt signalling can also activate atypical pathways such as Wnt/Ca2+, Wnt/JNK and other pathways, in which Wnt/Ca2 + plays an important role through calcium-dependent protein kinase, calmodulin and the transcription factor NFAT [21]; the Wnt/JNK signalling pathway activates JNK thr ough DSH, migrates into the nucleus, and regulates the activities of transcription factors c-Jun, Atf2, ELK1, DPC4, p53, etc. The activation of these atypical pathways also plays an important role in EMT and tumour invasion and metastasis [22]. We hypothesized that the Wnt/β-catenin signaling pathway and EMT cross-talk enhanced tumor drug resistance. then co-ip results showed that a trimer which form by TCF4(a key protein in WNT pathway), β-catenina and TWIST(a key protein in EMT) promote the development of RCC resistance. Studies have shown that RUNX3 forms a ternary complex with β-catenin/TCF4 to regulate Wnt signal transduction activity in intestinal tumors [23], TCF4/β-catenin complexes can regulate the progression of pulmonary hypertension [24], During EMT, TWIST interacts with β-catenin to enhance the transcriptional activity of the β-catenin/TCF4 complex [25], The activation of in colorectal cancer coupled with CD44 containing variable exon-6 (CD44v6) splicing in a positive feedback loop, thereby enhancing chemical resistance involved in multiple drug resistance [26].This is consistent with our hypothesis. In our study, we first constructed sunitinib-resistant RCC strains and Twist-overexpressing cells and then studied the changes in biological function and mechanisms by determining the protein expression levels of key nodes of the Wnt/β-catenin signalling pathway. After we found that sunitinib-resistant RCC was similar to Twist-overexpressing RCC strains in both function and protein expression levels, we further studied the interactions between them and verified the upstream and downstream genes in the Wnt/β-catenin signalling pathway and EMT pathway. Finally, we verified the findings in an animal model through zebrafish experiments.In addition, in the case of drug resistance and Twist overexpression, the continued use of normal doses of sunitinib still has a certain effect on inhibiting tumour growth and metastasis. The mechani sm may need further experiments to elucidate.

### Electronic supplementary material

Below is the link to the electronic supplementary material.

Supplementary Material 1

Supplementary Material 2

## Data Availability

The datasets used and/or analysed during the current study available from the corresponding author on reasonable request.
