# Supplementary material for: Interaction between Wnt/β-catenin signaling pathway and EMT pathway mediates the mechanism of sunitinib resistance in renal cell carcinoma
Source: BMC Cancer. 2024 Feb 5;24:175. doi: 10.1186/s12885-024-11907-5 (PMC10840195; doi:10.1186/s12885-024-11907-5)

Fig5 A

1. β-actin：42KDa

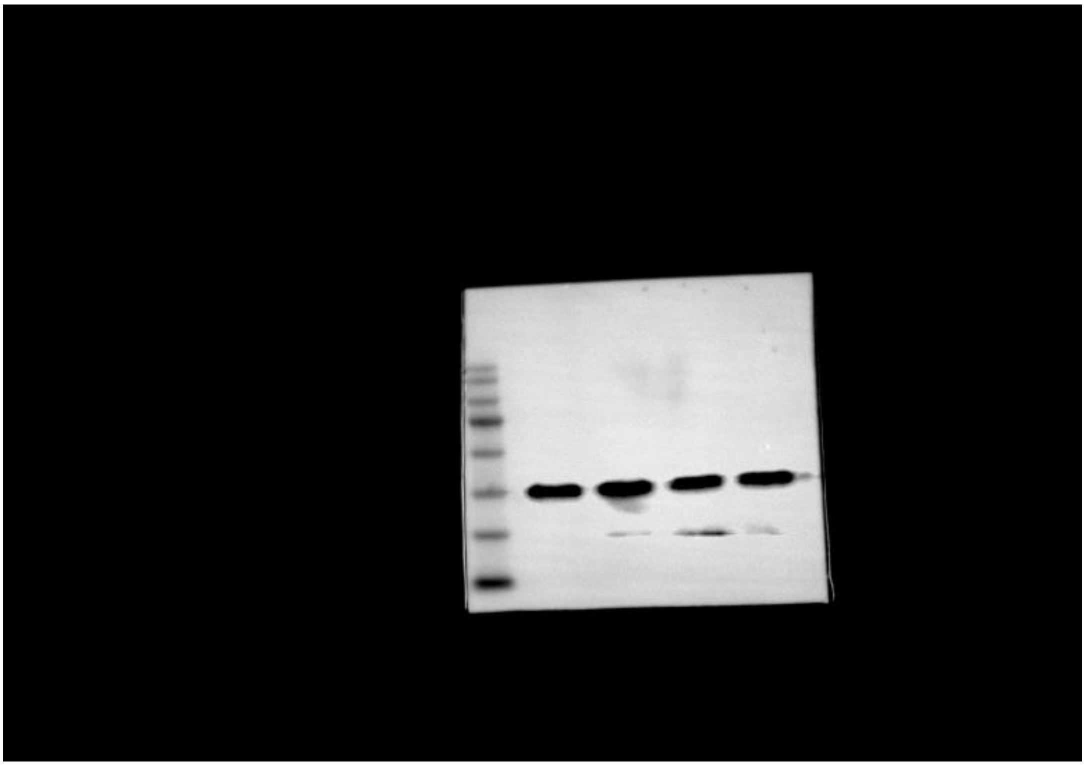

2. GSK3β：46 kDa

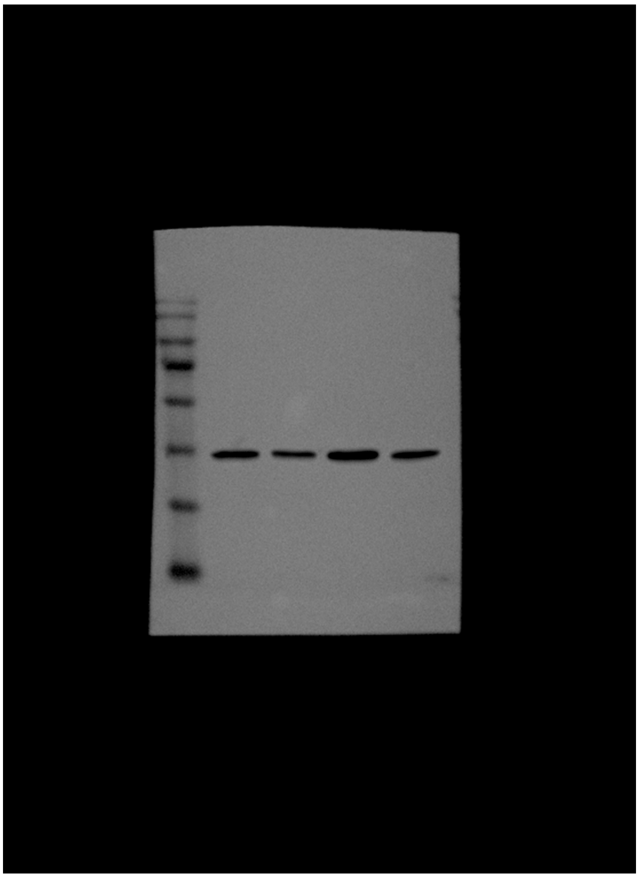

3. β-Catenin：85 kDa

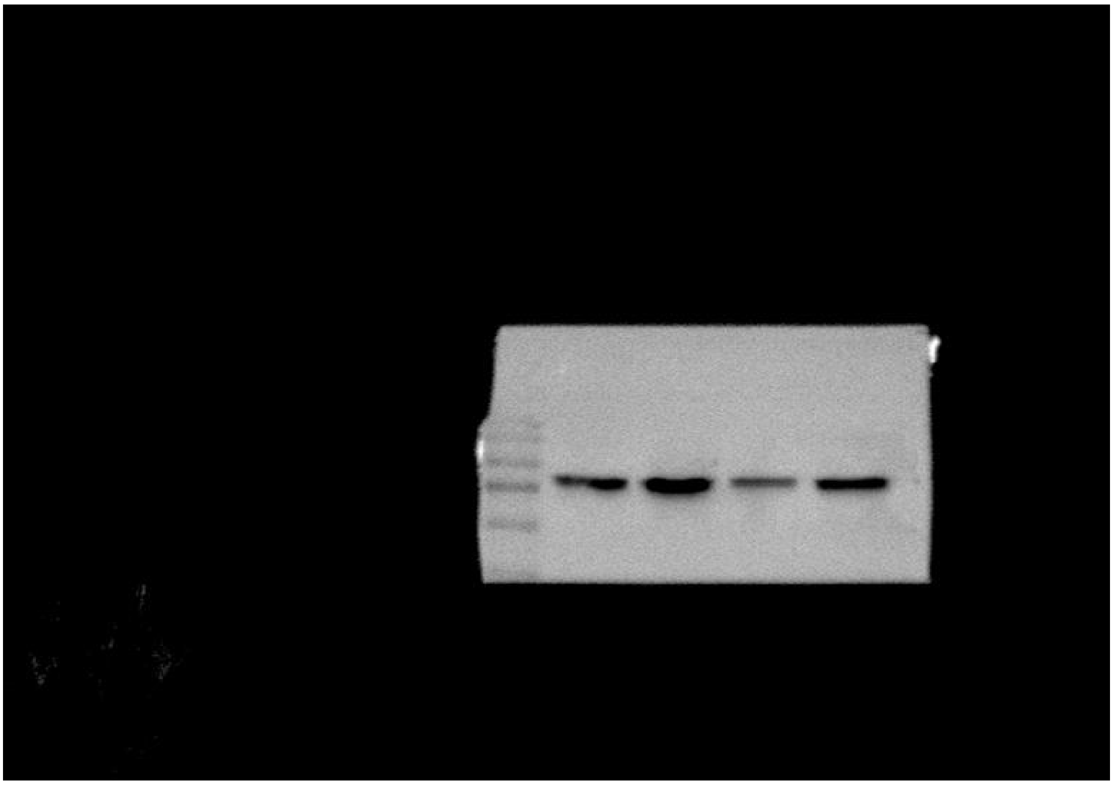

4. TCF4：90 kDa

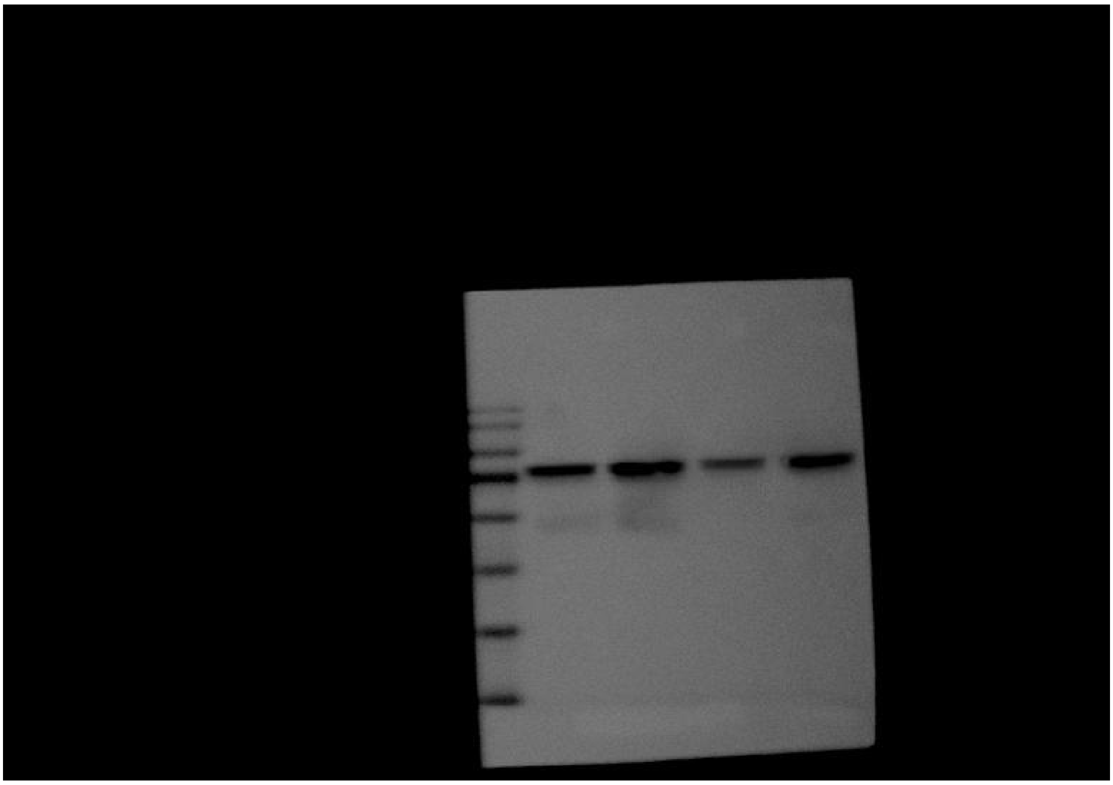

5. Snail： 29 kDa

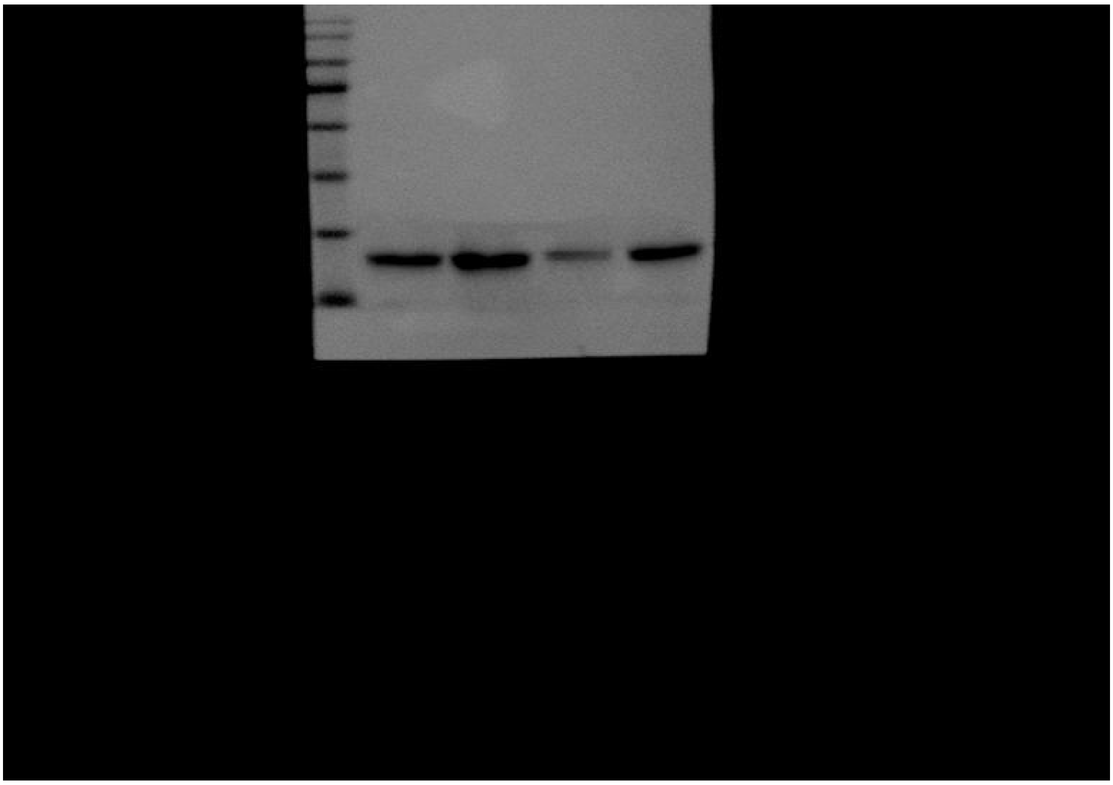

6. E-caderin：100Kda

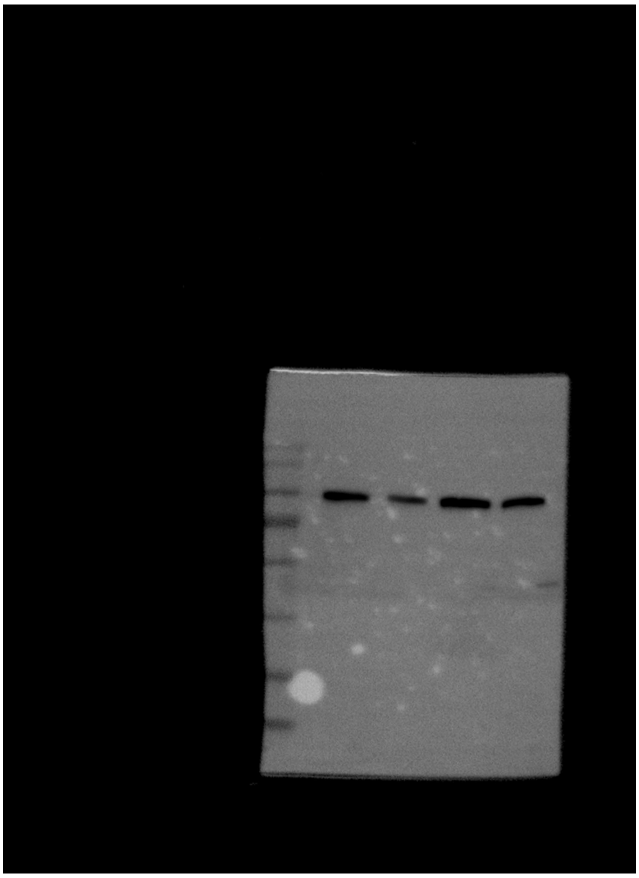

7. Twist：21 kDa

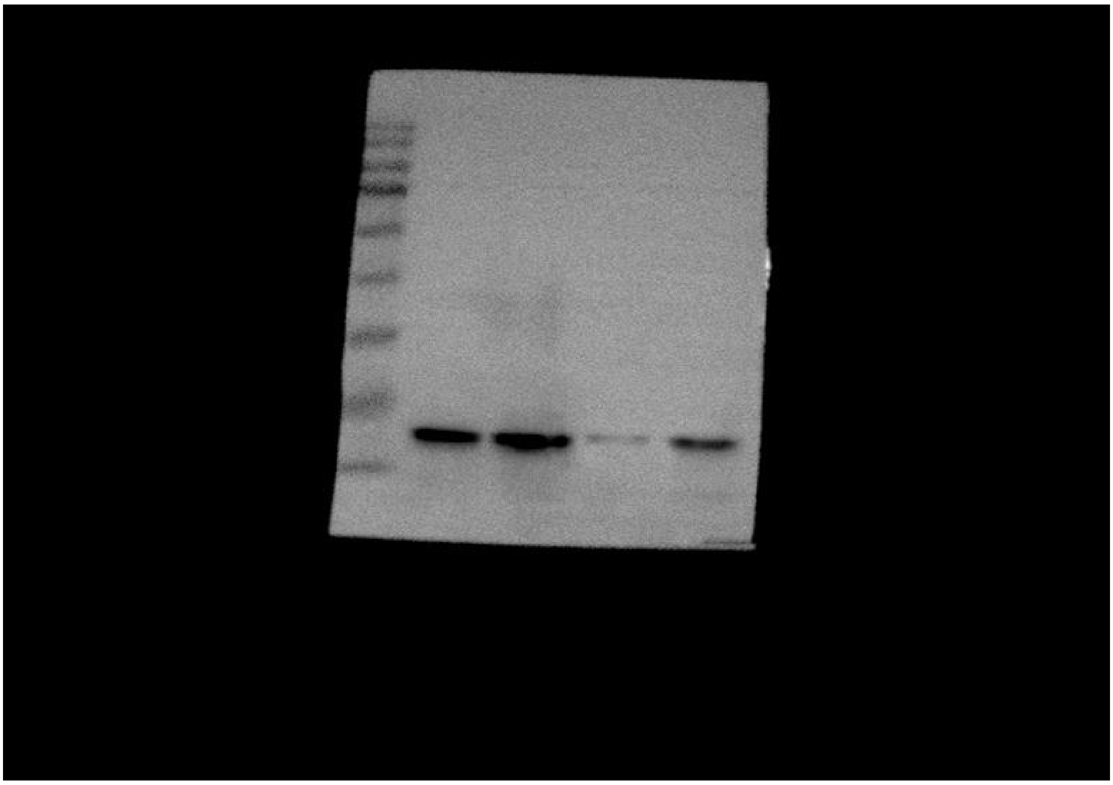

Fig6 A

1. β-actin：42KDa

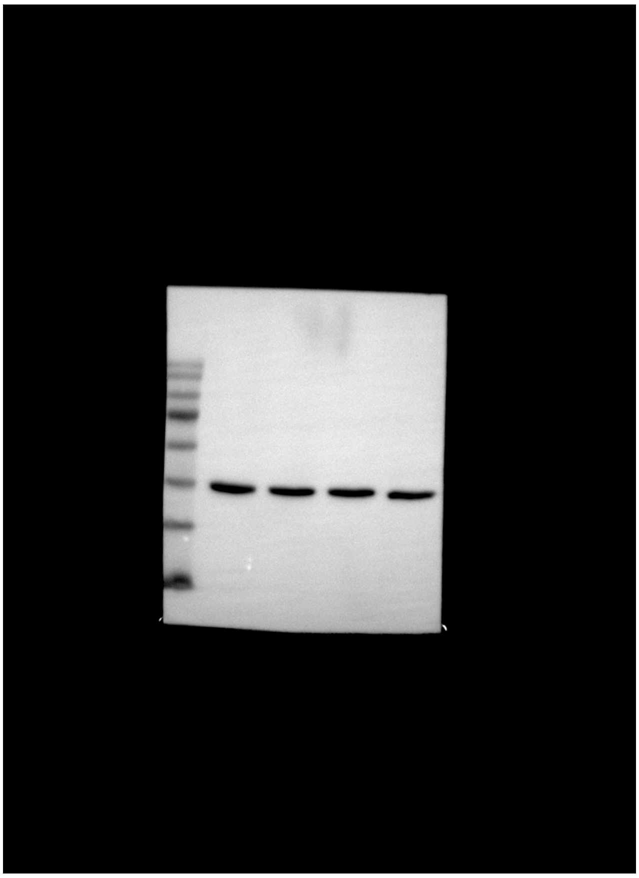

2. GSK3β：46 kDa

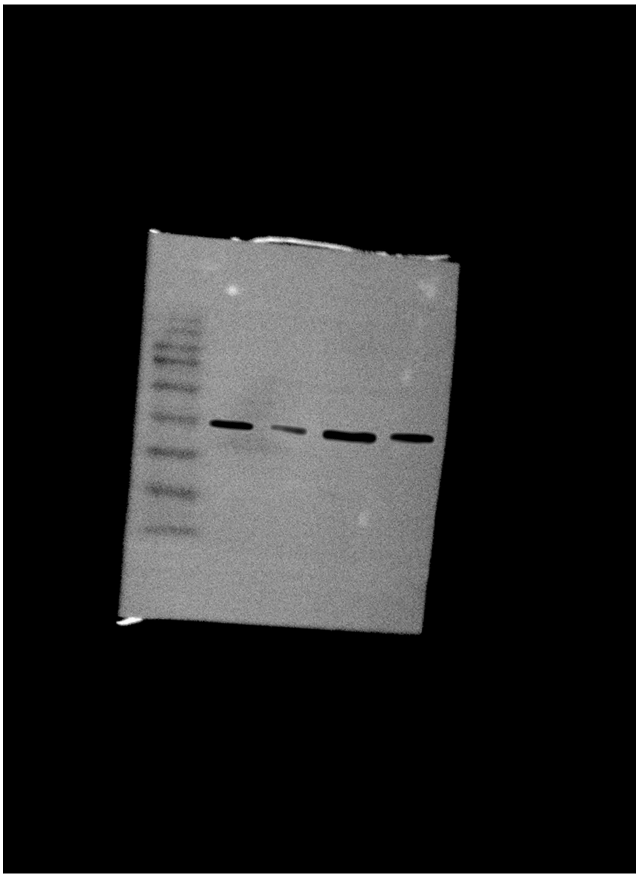

3. β-Catenin：85 kDa

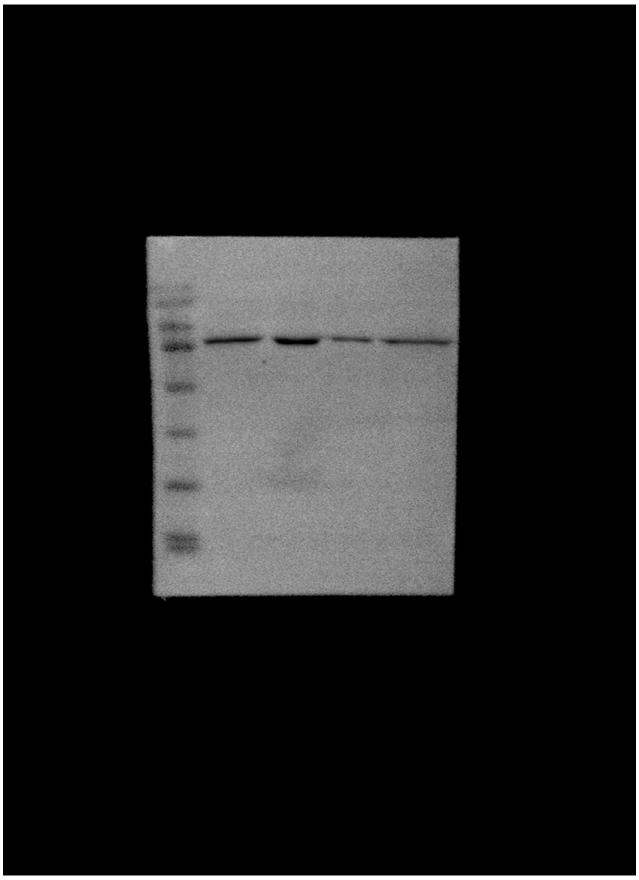

4. TCF4：90 kDa

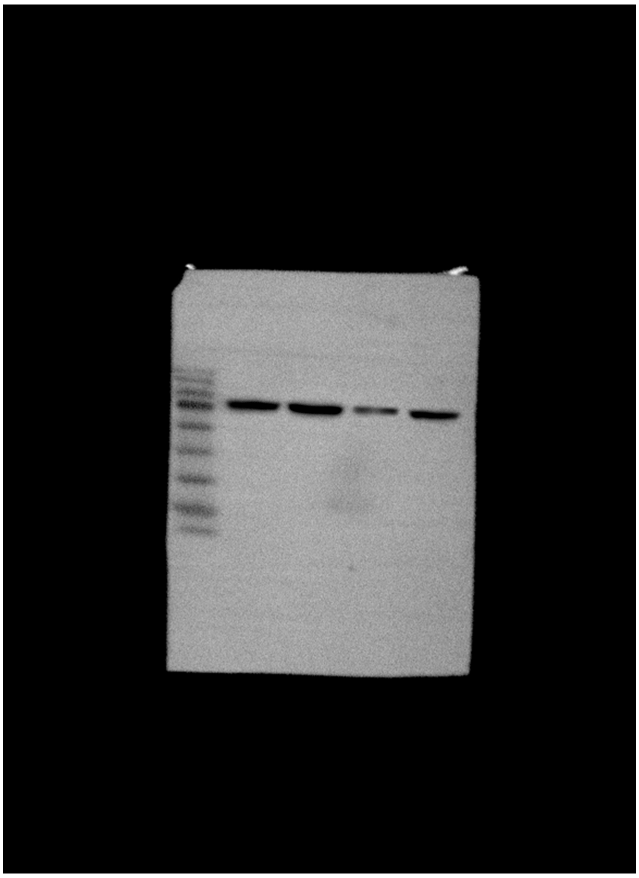

5. Snail： 29 kDa

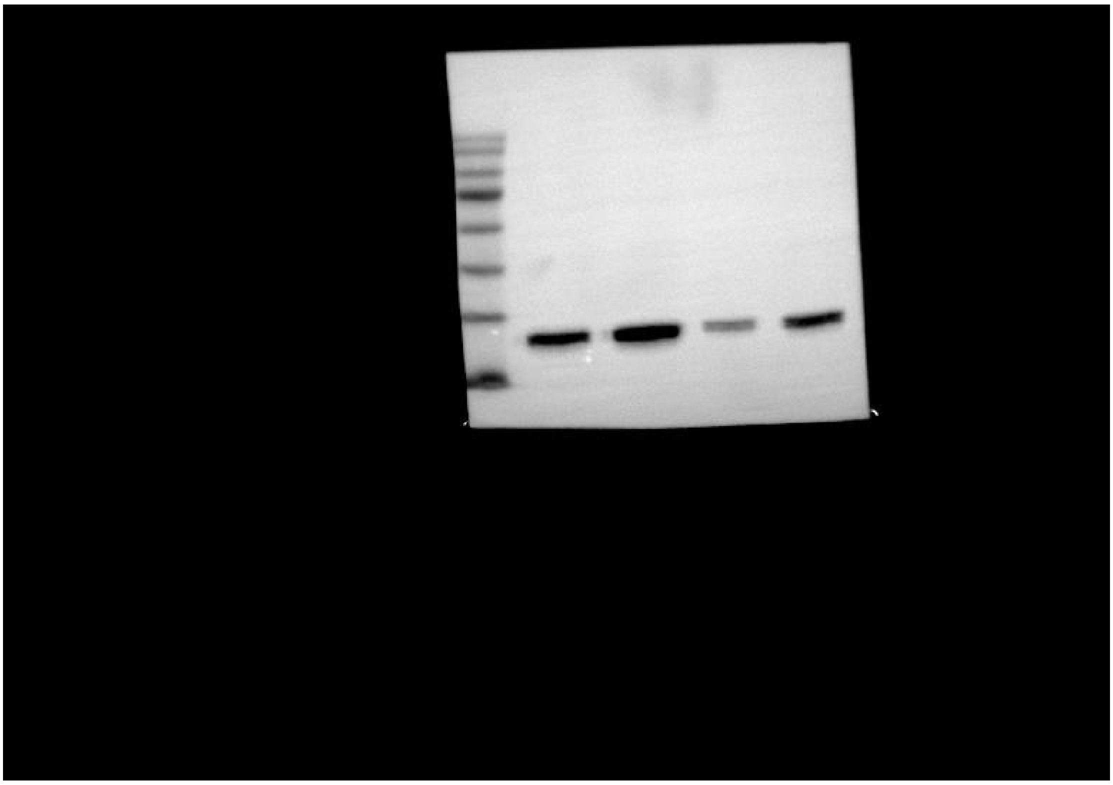

6. E-caderin：100Kda

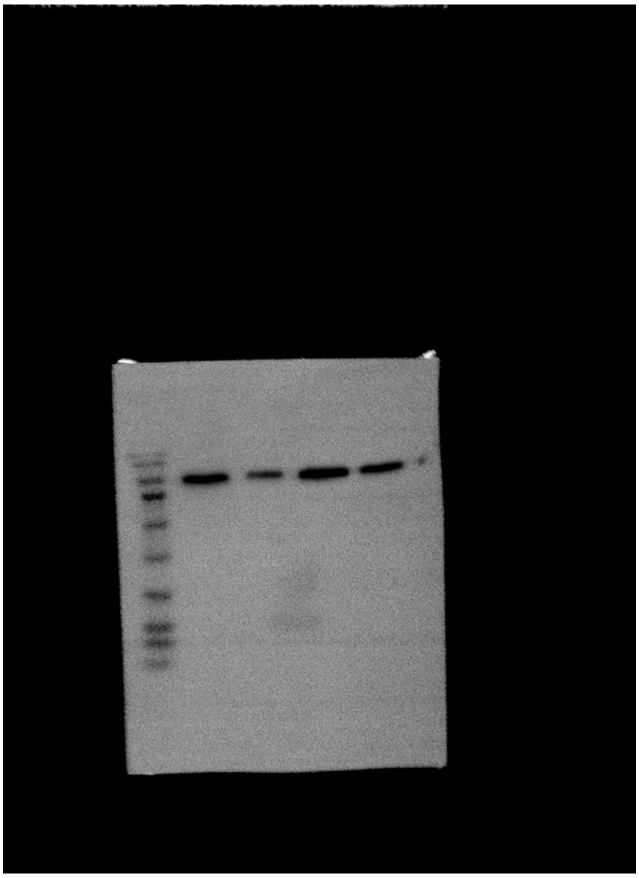

7. Twist：21 kDa

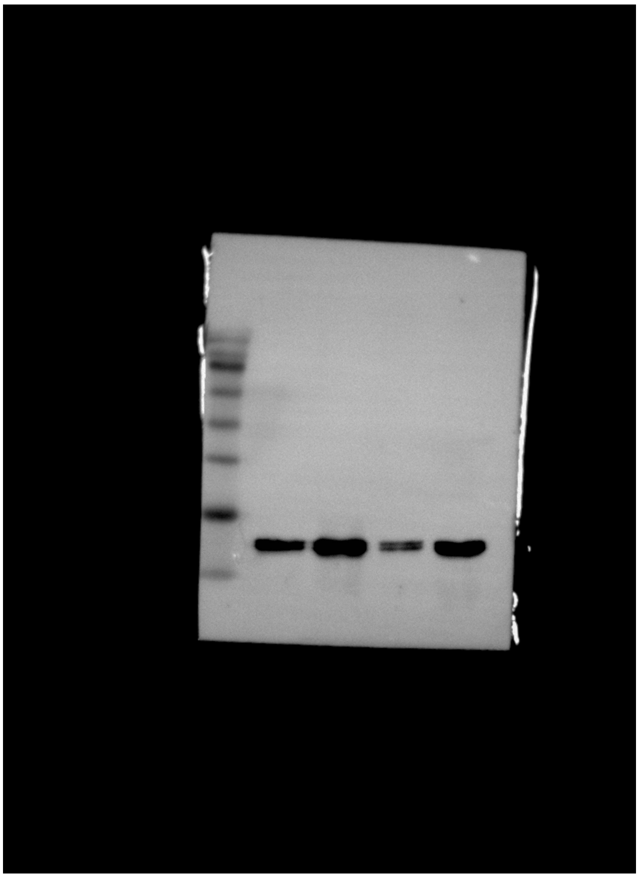

Fig 7 left

1. Twist：21 kDa

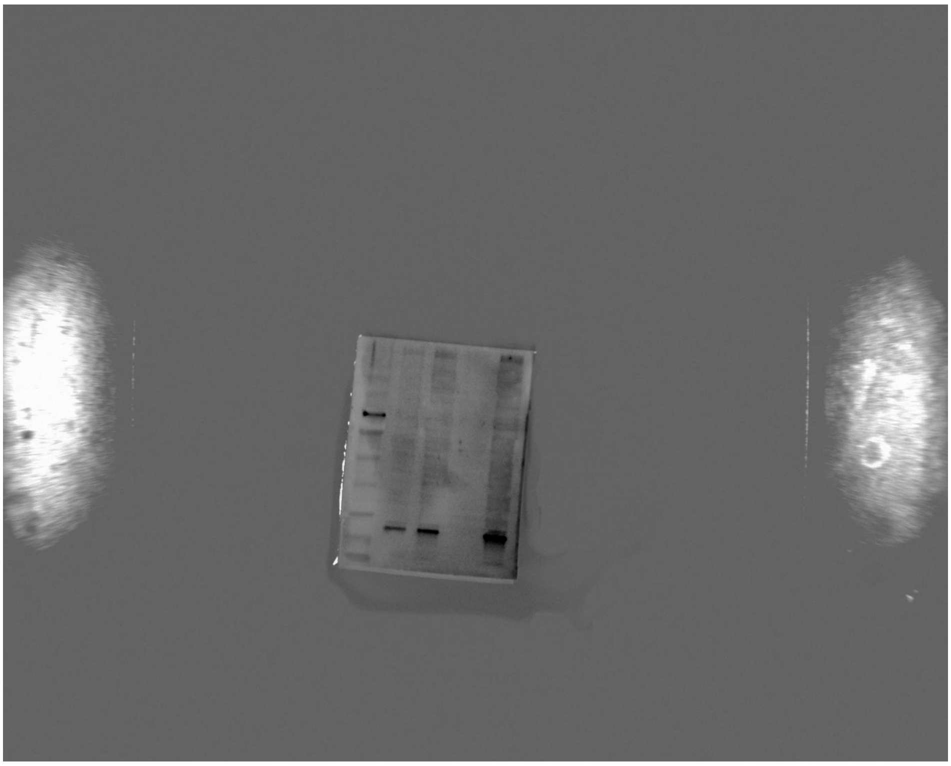

2. β-Catenin：85 kDa

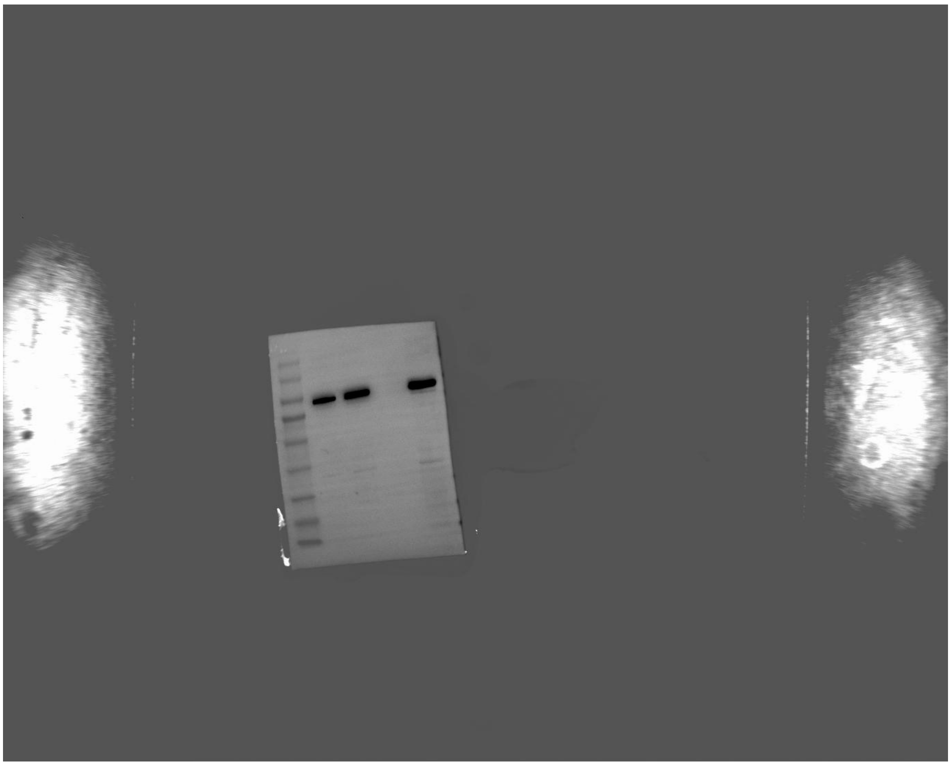

3.TCF4：90 kDa

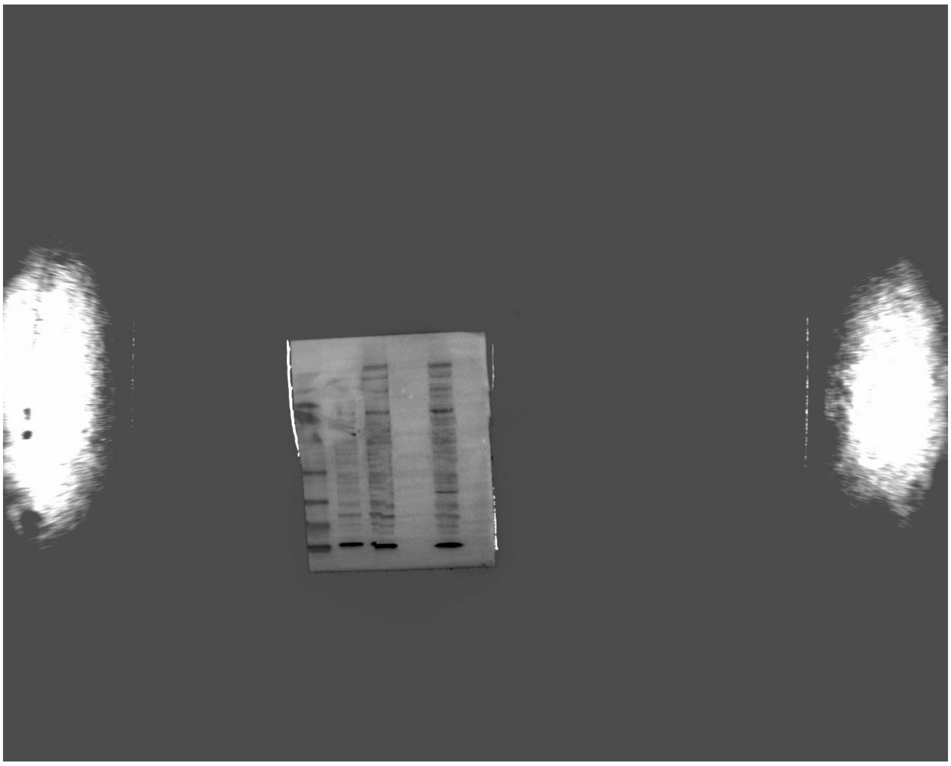

Fig 7 right

1. Twist：21 kDa

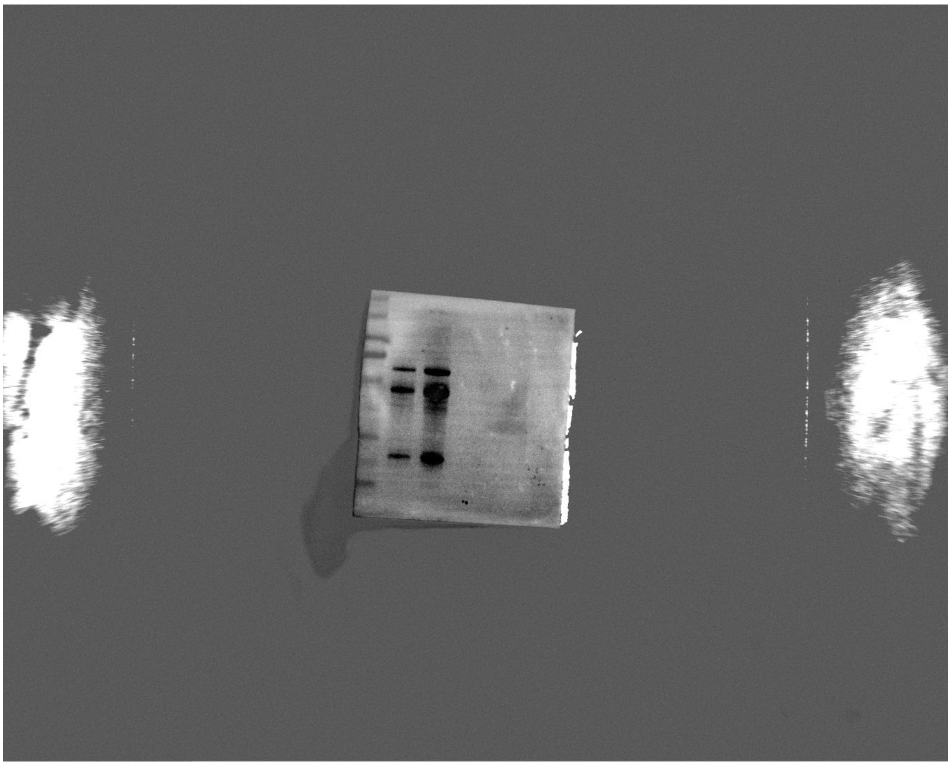

2. β-Catenin：85 kDa

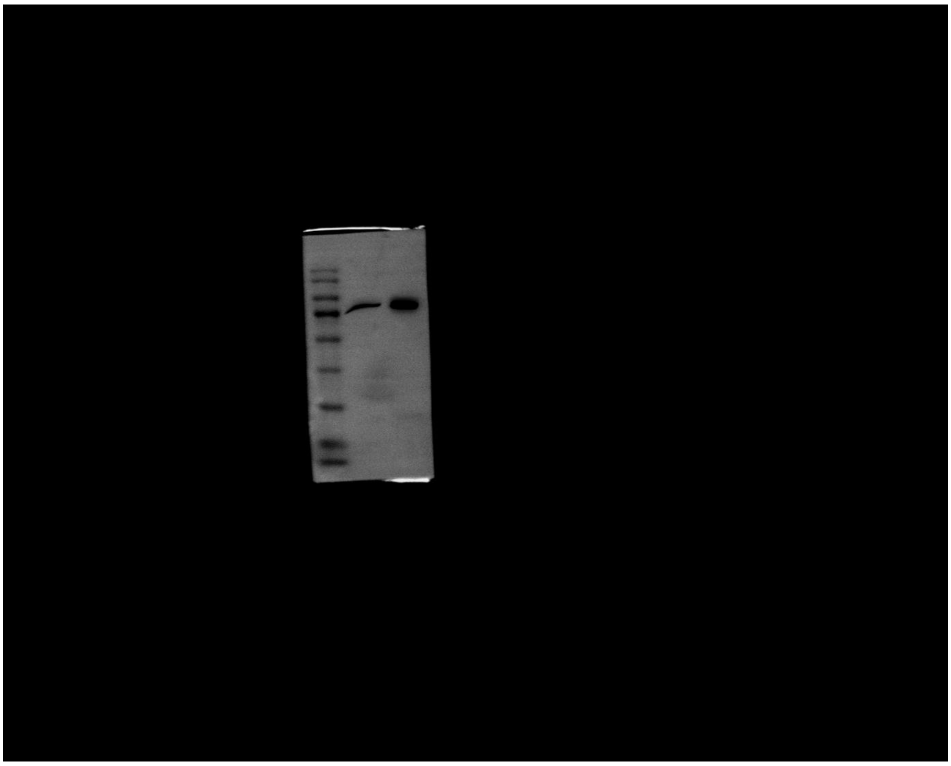

3.TCF4：90 kDa

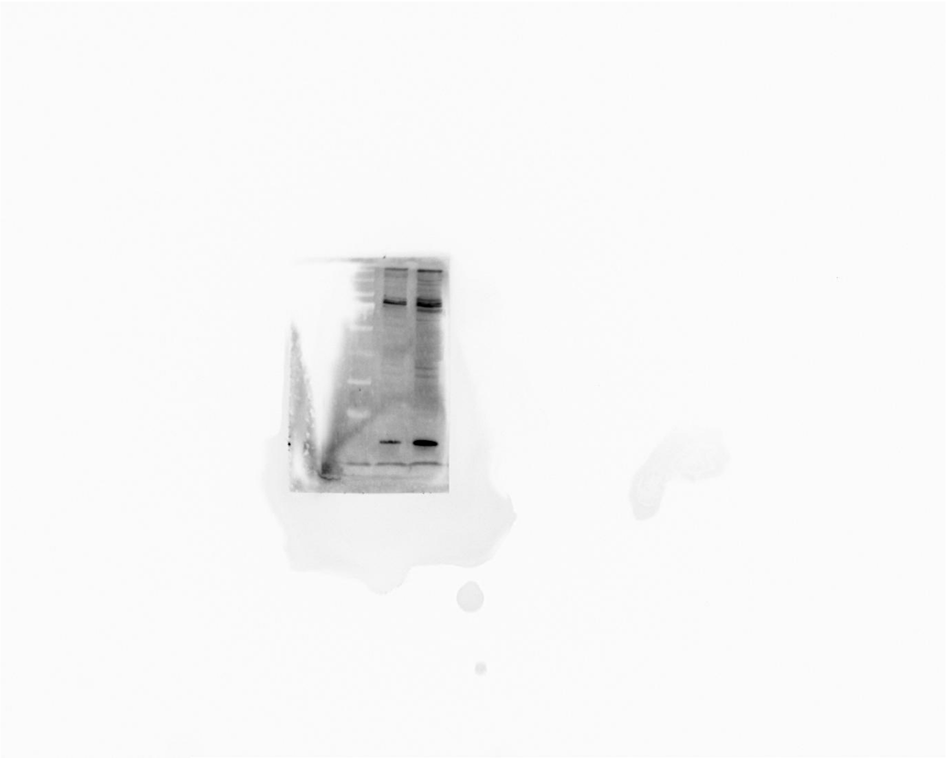

4. actin:42kDa

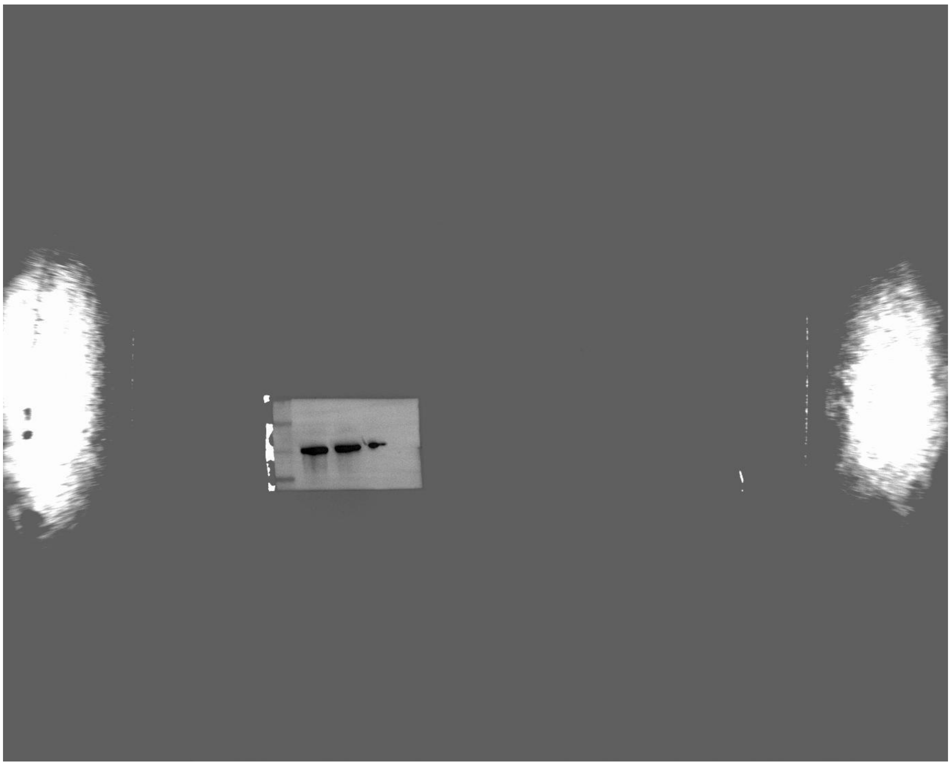

Supplement: Supplementary file 1 — Supplementary Material 1 [file 12885_2024_11907_MOESM1_ESM.pdf]
