# Supplementary figures and images for: Interaction between Wnt/β-catenin signaling pathway and EMT pathway mediates the mechanism of sunitinib resistance in renal cell carcinoma
Source: BMC Cancer. 2024 Feb 5;24:175. doi: 10.1186/s12885-024-11907-5 (PMC10840195; doi:10.1186/s12885-024-11907-5)

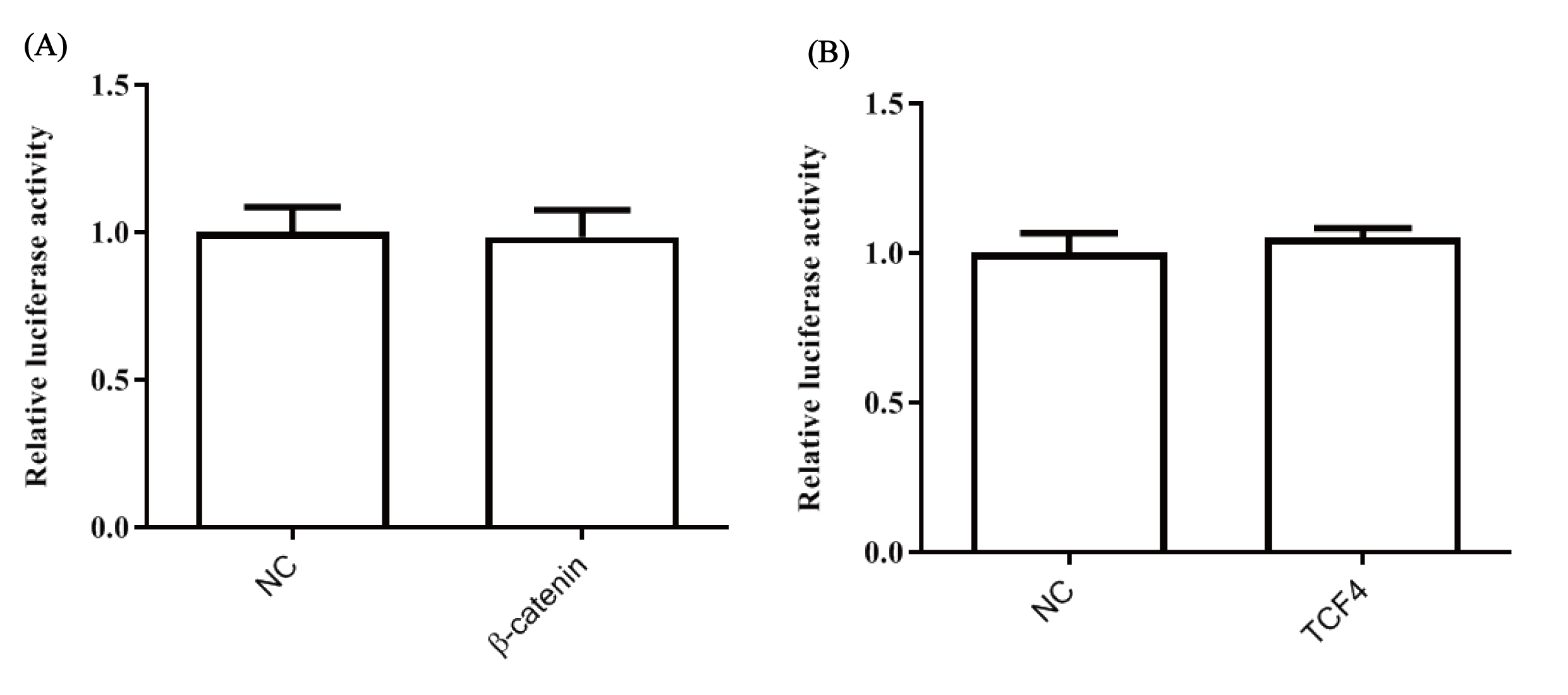

Supplement: Supplementary file 2 — Supplementary Material 2 [file 12885_2024_11907_MOESM2_ESM.tif]
